# A Spatially Detailed Model of Isometric Contraction Based on Competitive Binding of Troponin I Explains Cooperative Interactions between Tropomyosin and Crossbridges

**DOI:** 10.1371/journal.pcbi.1004376

**Published:** 2015-08-11

**Authors:** Sander Land, Steven A. Niederer

**Affiliations:** Department of Biomedical Engineering, King’s College London, United Kingdom; University of Michigan, UNITED STATES

## Abstract

Biophysical models of cardiac tension development provide a succinct representation of our understanding of force generation in the heart. The link between protein kinetics and interactions that gives rise to high cooperativity is not yet fully explained from experiments or previous biophysical models. We propose a biophysical ODE-based representation of cross-bridge (XB), tropomyosin and troponin within a contractile regulatory unit (RU) to investigate the mechanisms behind cooperative activation, as well as the role of cooperativity in dynamic tension generation across different species. The model includes cooperative interactions between regulatory units (RU-RU), between crossbridges (XB-XB), as well more complex interactions between crossbridges and regulatory units (XB-RU interactions). For the steady-state force-calcium relationship, our framework predicts that: (1) XB-RU effects are key in shifting the half-activation value of the force-calcium relationship towards lower [Ca^2+^], but have only small effects on cooperativity. (2) XB-XB effects approximately double the duty ratio of myosin, but do not significantly affect cooperativity. (3) RU-RU effects derived from the long-range action of tropomyosin are a major factor in cooperative activation, with each additional unblocked RU increasing the rate of additional RU’s unblocking. (4) Myosin affinity for short (1–4 RU) unblocked stretches of actin of is very low, and the resulting suppression of force at low [Ca^2+^] is a major contributor in the biphasic force-calcium relationship. We also reproduce isometric tension development across mouse, rat and human at physiological temperature and pacing rate, and conclude that species differences require only changes in myosin affinity and troponin I/troponin C affinity. Furthermore, we show that the calcium dependence of the rate of tension redevelopment k_tr_ is explained by transient blocking of RU’s by a temporary decrease in XB-RU effects.

## Author Summary

Force generation in cardiac muscle cells is driven by changes in calcium concentration. Relatively small changes in the calcium concentration over the course of a heart beat lead to the large changes in force required to fully contract and relax the heart. This is known as ‘cooperative activation’, and involves a complex interaction of several proteins involved in contraction. Current computer models which reproduce force generation often do not represent these processes explicitly, and stochastic approaches that do tend to require large amounts of computational power to solve, which limit the range of investigations in which they can be used. We have created an new computational model that captures the underlying physiological processes in more detail, and is more efficient than stochastic approaches, while still being able to run a large range of simulations. The model is able to explain the biological processes leading to the cooperative activation of muscle. In addition, the model reproduces how this cooperative activation translates to normal muscle function to generate force from changes in calcium across three different species.

## Introduction

Tension generation in cardiac muscle is a highly cooperative process, with significant increases in tension caused by relatively small increases in the calcium concentration. The Hill coefficient (*n*_*H*_) describing the degree of cooperativity of the force-calcium relationship is typically around *n*_*H*_ = 3 in experiments on skinned muscle cells [1], and as high as *n*_*H*_ = 10 in intact cells [2]. Our understanding of the molecular mechanisms giving rise to this cooperative activation and the precise regulation of tension generation required for effective cardiac pump function remains incomplete. However, there is a general agreement on the potential types of interactions involved in cooperative activation between regulatory units (RU) and crossbridges (XB) [3–5]. Each half-sarcomere in a myocyte contains 26 RU’s, and each RU consists of 7 actin monomers, one long tropomyosin molecule spanning the actin monomers, and a complex of troponin (troponin I, troponin C and troponin T) which regulates local activation. Within an RU, calcium (Ca^2+^) bind to troponin C (TnC), causing a conformational change in tropomyosin, unblocking actin for myosin crossbridge (XB) binding [6].

Underlying cooperative activation, three types of interactions are proposed between regulatory units and crossbridges. Cooperative effects between RU’s are known as ‘RU-RU cooperativity’, where unblocking of tropomyosin in one RU leads to an increased probability of unblocking in a nearby RU, due to overlap of tropomyosin molecules between neighbouring RU’s. Evidence in support of these effects includes experimental data which shows a significant decrease in cooperativity when the overlap between neighbouring tropomyosin units is removed or reduced [7–9], dependence on nearest neighbour interactions in cardiac muscle [10], and modifications to long-range cooperativity by phosphorylation of tropomyosin [11]. In addition there are cooperative interactions in which the binding of crossbridges increases the rate at which further crossbridges bind, known as ‘XB-XB cooperativity’ [12, 13]. Although XB-XB interactions can increase the steady-state force per activated RU, more complex interactions with neighbouring RU’s are involved in their effect on cooperativity. These more complex interactions by which crossbridges affect RU activation are known as ‘XB-RU cooperativity’ [13, 14]. Evidence for the importance of these effects on muscle activation can be seen from various experiments in which calcium sensitivity is affected by changes to crossbridge affinity using crossbridge inhibitors and enhancers [1, 15–17]. A potential factor in XB-RU cooperativity are the proposed effects of tension generation on the affinity of TnC for Ca^2+^ [18, 19]. The mechanisms and significance of this interaction remain controversial, with some researchers claiming this effect appears mainly from non-physiological rigor crossbridges [1], while others point to it as a key component of normal muscle function [18, 20, 21].

**Fig 1:**
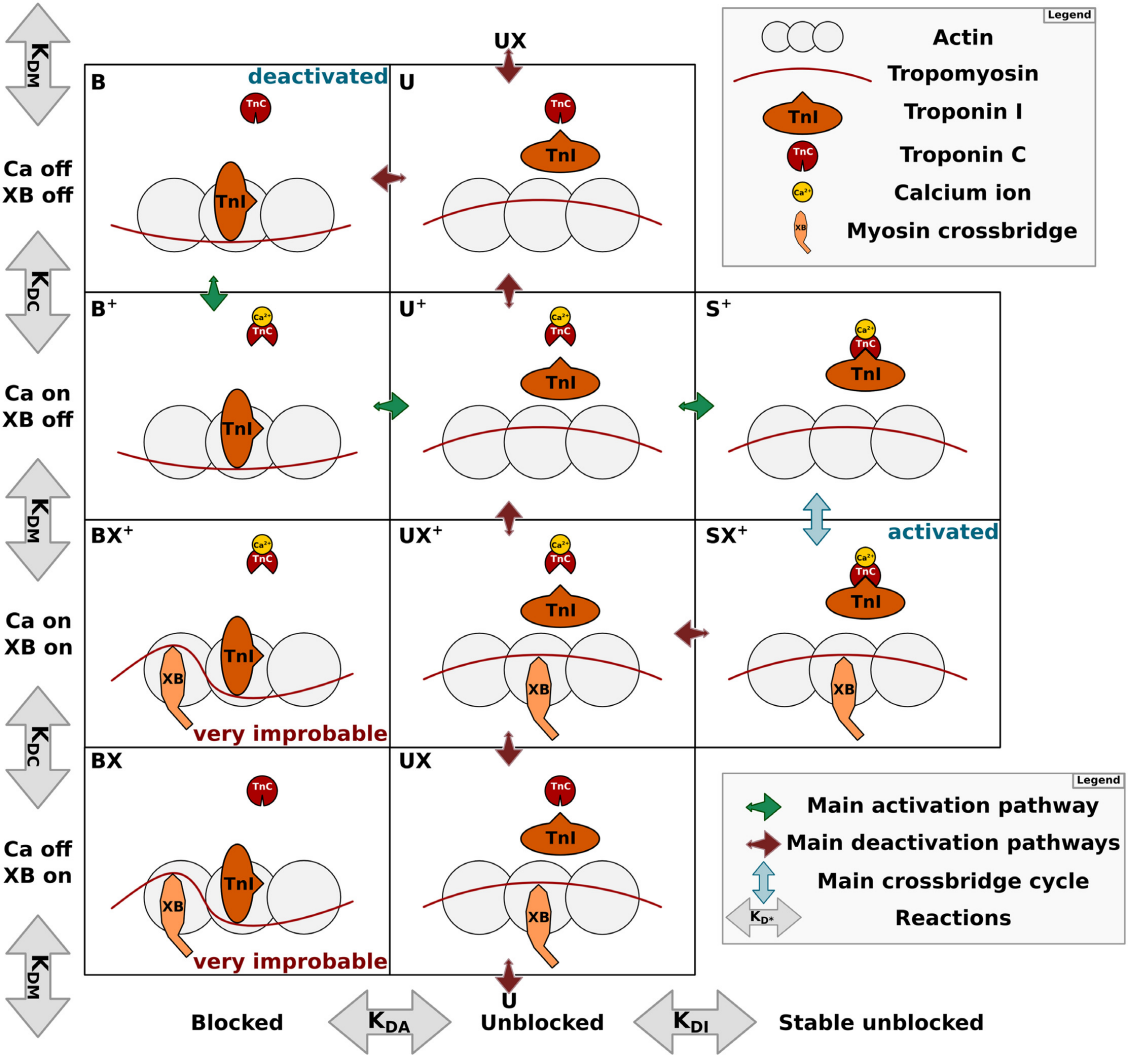
Overview of RU states. Each regulatory unit (RU) along the actin-tropomyosin thin filament contains troponin C, which binds calcium, and troponin I, which can bind either to actin or troponin C. These reactions, in addition to the binding of crossbridges, define each of the states. In the schematic, state names with + have calcium bound, state names which include an ‘X’ have crossbridge(s) bound, and B, U, S refer to the labels ‘blocked’, ‘unblocked’, ‘stable unblocked’ at the bottom of the schematic. ‘Blocked’ refers to troponin I bound to actin, which blocks myosin binding. ‘Unblocked’ refers to troponin I being not bound to actin, and ‘stable unblocked’ refers to troponin I being held in place by troponin C. Each state allows for crossbridge binding, although this is very improbable in the ‘blocked’ states, such that states (‘BX’ and ‘BX^+^’) rarely occur. Note that all transitions between neighbouring states exist, in addition to transitions between the top and bottom rows. The green arrows indicate the main pathway during activation, with Ca^2+^ binding to TnC, and TnI moving from actin to TnC⋅Ca^2+^ to allow crossbridge binding. Red arrows indicate the main deactivation pathway, TnI detaching from TnC⋅Ca^2+^, followd by Ca^2+^ detaching from TnC and TnI binding to actin to block crossbridge binding.

In addition to uncertainties in the biophysical basis for cooperativity, the exact link between calcium binding to TnC and the movement of tropomyosin has remained obscure, troponin I (TnI) is known to play a key role in transmitting this signal [22–24], and in recent years this link has been clarified with research on crystal structures of troponin [25, 26]. These studies showed that calcium binding to TnC opens up a hydrophobic patch on TnC which has a high affinity for the switch region of TnI [27]. The movement of the switch region also moves the nearby inhibitory (‘C-terminal’) region of TnI which is responsible for pinning tropomyosin in the blocking position on actin in resting muscle [28]. The competitive binding of these TnI regions to both TnC and actin results in the unblocking of actin at higher Ca^2+^ concentration, allowing myosin crossbridges to bind and generate force. Further support for the critical role of TnI is given by its numerous phosphorylation sites and role in regulating muscle function through *β*-adrenergic stimulation and the response to length-dependent activation [28–30]. Fig 1 gives an overview of a regulatory unit (RU) and its states in this competitive binding framework.

In the three-state framework proposed by McKillop and Geeves [31] RU’s can be either in the ‘blocked’ state with TnI pinning tropomyosin to actin, in the neutral ‘closed’ state where myosin crossbridges are able to bind, or in the ‘open’ state with crossbridges having moved tropomyosin in the opposite direction compared to TnI binding. The continuous flexible chain models represent the spatial deformation of tropomyosin along the whole thin filament. At points along the chain with a TnI binding site or crossbridge, the chain is in a fully ‘blocked’ or ‘open’ position respectively. However, in the space between bound sites, the chain can occupy a continuum of intermediate states. Due to the single TnI binding site per RU, we can still unambiguously refer to an RU as blocked based on TnI binding. Describing an RU itself as ‘open’ becomes more problematic in this modelling framework, as there are 2–3 crossbridges per RU and any combination of these can be bound to actin at any one time. In the rest of this paper we refer to the state of RU’s only as ‘blocked’ and ‘unblocked’ based on TnI-actin binding, regardless of the tropomyosin deformation induced or number of crossbridges bound near the RU.

There are several challenges in applying these advances in physiology to create a computational model of cardiac contraction that is both tractable for a wide range of simulation and analysis, and captures the critical physiological features of the underlying proteins. Firstly, computational models which include tension-dependent feedback mechanisms often suffer from non-physiological hysteresis, in which tension generation is higher for decreasing calcium compared to increasing calcium [32]. Secondly, in the absence of a clear mechanistic explanation for cooperativity, computational models based on ordinary differential equations (ODE) tend to use phenomenological representation of cooperativity to achieve adequate tension development [5, 33–36]. Some recent developments have begun to address these shortcomings, including detailed models of the thin filament based on spatial interaction of tropomyosin [37, 38]. Firstly, the work by Campbell et al. includes a model of tropomyosin interaction between neighbouring RU [38], and is based on ODEs. However, it is limited to approximately 9 RU’s, and requires the assumption that calcium bound to TnC does not unbind in the tropomyosin ‘closed’ state. Extending the model beyond these assumptions quickly leads to an increase in the required number of states beyond what is computationally tractable. Nevertheless, the model is arguably the most biophysically detailed contraction model to have been applied in the context of a whole-organ cardiac mechanics [39]. Secondly, a more detailed underlying model of cooperativity is given by models of tropomyosin as a continuous flexible chain, based on the work by Smith et al. [40–43]. These approaches assume that tropomyosin, which consists of many molecules overlapping end-to-end to form a long filament, can be modelled as a homogeneous flexible chain. The deformation of the tropomyosin chain in these models is determined by a combination of weak electrostatic interactions with actin and elastic deformation of the chain. Although still a simplification that ignores potential inhomogeneities arising from end-to-end overlap, these models provide a more detailed description of tropomyosin kinetics which are able to describe longer range interactions in the thin filament, compared to models which assume only nearest-neighbour interactions. Solving these more detailed models remains computationally challenging, and results are typically given by developing approximations for the equations of the deformation of tropomyosin, or applying stochastic approaches to predict a steady-state force-calcium relationship.

Our goal in this article is to create an ODE-based model of cardiac contraction with a biophysically detailed representation of cooperativity based on the competitive binding model of troponin I and the continuous flexible chain model for tropomyosin. The formulation of an ODE-based model facilitates modelling of a wide range of simulations of dynamic function of muscle, and will allow us to link this model to whole organ mechanics in the future.

This paper is organized as follows: We start with a general theory on modelling tropomyosin as a continuous flexible chain and the use of Boltzmann’s law. The section “Steady-state models” describe our model for the steady-state blocking and unblocking of RU’s in the absence of myosin crossbridges. We extend this model to include myosin crossbridges, and develop techniques to make this approach computationally tractable. This extended steady-state model is then used to explain the sources of cooperativity, and the effects on myosin binding in producing XB-RU effects and changes in Ca^2+^-TnC affinity. The section “Dynamic models” develops the dynamic models of cardiac contraction, which we use to investigate the role of cooperative activation in isometric tension development across different species, as well as the influence of cooperative effects on the rate of tension redevelopment and its dependence on Ca^2+^.

## Models

Our framework combines a model of the deformation of the tropomyosin filament with the more typical protein-protein interactions. This is accomplished using Boltzmann’s law, which says that given a system of molecules with different states *S*_1_, *S*_2_, … *S*_*n*_ with corresponding energies *E*_1_, *E*_2_, … *E*_*n*_, the probability *P*(*S*_*i*_) of being in a state that has energy *E*_*i*_ when the system is in thermal equilibrium is

$$P\left(S_{i}\right)∼e^{-\frac{E_{i}}{k_{\;B}T}}$$ (1)

where *k*_*B*_ is Boltzmann’s constant and *T* the absolute temperature in Kelvin [44].

For our model, we consider interactions between TnI, TnC, Ca^2+^, actin, and myosin and the deformation of tropomyosin to be the significant interactions [43]. We introduce four constants to represent differences in free energy related to the different protein-protein interactions that regulate cooperative tension development: *E*_*C*_ is the energy required for Ca^2+^ binding to TnC to form TnC⋅Ca^2+^, *E*_*A*_ is the energy required for TnI binding to actin to form TnI⋅A, *E*_*I*_ is the energy required for TnI binding to TnC⋅Ca^2+^ to form TnI⋅TnC⋅Ca^2+^, and *E*_*M*_ is the energy required for myosin binding to actin. For example, *E*_*A*_ is the difference in free energy between the state with TnI bound to actin (TnI⋅A) and unbound from actin (TnI+A). This constant can be linked to the ratio between occupation of the states and the dissociation constant *K*_DA_ via the Boltzmann term:

$$e^{-\frac{E_{A}}{k_{B}T}}=e^{-\frac{\left(E_{\text{TnI}\cdot \text{A}}-E_{\text{TnI}+\text{A}}\right)}{k_{B}T}}=\frac{P(\text{TnI}\cdot \text{A})}{P(\text{TnI}+\text{A})}=\frac{1}{K_{\text{DA}}}$$ (2)

Our model assumes that binding of TnI to TnC in the absence of Ca^2+^ is improbable enough to be negligible, as the hydrophobic patch on TnC is not opened. In addition this implies that the unbinding of Ca^2+^ from TnI⋅TnC is similarly negligible, because of thermodynamic consistency with the high energy required to form TnI⋅TnC in the absence of Ca^2+^. This assumption leads to the absence of the states ‘S’ and ‘SX’ in Fig 1 and the corresponding transitions (‘UX → SX’ and ‘SX^+^ → SX’). However, note that the unbinding of TnI from TnC and subsequent unbinding of Ca^2+^ is still possible in unblocked RU’s, it simply leaves TnI bound to neither actin nor TnC as in the unblocked states (middle column) in Fig 1. Likewise, TnI can unbind from actin, leading to unblocking of RU’s and even binding of myosin crossbridges in the absence of Ca^2+^, corresponding to the transitions B → U → UX in Fig 1. This is improbable under normal circumstances, but has been observed experimentally in conditions of low ATP [45].

Unlike similar stochastic frameworks in which each transition can be handled separately [43], for our ODE-based approach we combine all components to give the total free energy of a half-sarcomere. Combined with a model which gives the free energy associated with tropomyosin deformation *E*_tm_, this total free energy is given by:

$$E_{\text{tot}}\left(i,j,k,l\right)=E_{\text{tm}}+\left(n-i\right)E_{A}+jE_{M}+k\left(E_{I}+E_{C}\right)+lE_{C}$$ (3)

Where *n* is the number of RU’s, and

- *i* = *N*_*u*_(tm) the number of unblocked RU’s for a tropomyosin state tm (and thus (*n* − *i*) the number of blocked RU’s),
- *j* = *N*_xb_(tm) the total number of crossbridges bound for a tropomyosin state tm,
- *k* is the number of RU’s with both Ca^2+^ and TnI bound to TnC (states S^+^, SX^+^ in Fig 1),
- *l* is the number of RU’s with Ca^2+^, but not TnI bound to TnC (states B^+^, BX^+^, U^+^, UX^+^).

We represent the energy related to tropomyosin deformation (*E*_tm_) using a continuous flexible chain model, which approximates all end-to-end connected tropomyosin molecules as a single chain. The displacement of tropomyosin is regulated by troponin complexes in 26 RU’s per half-sarcomere spaced 38.5 nm apart [46]. With respect to tropomyosin deformation, regulatory units can be either in the ‘blocked’ state with TnI pinning tropomyosin to actin at -25°, or in an ‘unblocked’ state resulting in an angle determined by neighbouring units, and tending towards the neutral 0° position. In addition, myosin crossbridges displace tropomyosin in the opposite direction to TnI, at +10°. The chain will assume a minimal energy configuration constrained by these ‘fixed points’ introduced by TnI and myosin. More formally, the deformation of this chain is represented by the angle *ϕ*(*x*) by which tropomyosin is displaced from its neutral position in the helical groove of the actin filament, and we solve the energy minimization problem:

$$\text{minimize}\;E_{\text{tm}}\left(\phi \left(x\right)\right)\;\text{with}\;\text{Dirichlet}\;\text{boundary}\;\text{conditions}\;\phi \left(x_{1}\right)=\phi_{1},\phi \left(x_{2}\right)=\phi_{2}\;\ldots$$

The boundary conditions represent stable points that force tropomyosin to have specific angles at specific locations along the actin filament introduced by troponin I (*ϕ*(*x*_*i*_) = −25°) and myosin binding (*ϕ*(*x*_*i*_) = +10°) [47, 48]. Solving this minimization problem results in the deformation *ϕ*(*x*) along with the free energy *E*_tm_ for a certain tropomyosin ‘state’ dependent only on these stable points. The full description of the equations and solution using a finite element model is given in the Supporting Information (S1 Text, S1 Fig).

As we are interested in the global properties of the thin filament which determine RU unblocking and force generation, rather than the probability of calcium being bound to any specific RU, we sum over all possibilities of Ca^2+^ or TnI being bound in RU’s, neither of which affect the deformation of tropomyosin. Specifically, for a tropomyosin state tm with *i* = *N*_*u*_(tm) unblocked RU’s, there are (ik) ways to have *k* TnI bound to TnC⋅Ca^2+^. In addition there are (n−kl) ways to have *l* Ca^2+^ bound to TnC without TnI being bound. This allows us to calculate the probability *P*(tm) of being in the thin filament state tm as:

$$\begin{array}{cc} P\left(\text{tm}\right) & ∼\overset{i}{\underset{k=0}{\sum }}\overset{n-k}{\underset{l=0}{\sum }}\left(\begin{array}{c} i\\ k \end{array}\right)\left(\begin{array}{c} n-k\\ l \end{array}\right)e^{-\frac{E_{\text{tot}}\left(i,j,k,l\right)}{k_{B}T}}\;\text{where}\;i=N_{u}\left(\text{tm}\right),j=N_{\text{xb}}\left(\text{tm}\right)\\ & =e^{-\frac{E_{\text{tm}}}{k_{B}T}}\frac{1}{K_{\text{DA}}^{n-i}}\frac{1}{K_{\text{DM}}^{j}}{\left(1+\frac{\left[{\text{Ca}}^{2+}\right]}{K_{\text{DC}}}\right)}^{n-i}{\left(1+\frac{1}{K_{\text{DI}}}\frac{\left[{\text{Ca}}^{2+}\right]}{K_{\text{DC}}}+\frac{\left[{\text{Ca}}^{2+}\right]}{K_{\text{DC}}}\right)}^{i} \end{array}$$ (4)

Where the equality follows by applying the binomial theorem (for details see S1 Text) and the relation between the energies and dissociation constants (see Table 1). This result can also be understood more intuitively by considering there are *n* − *i* RU’s which have nothing bound, or Ca^2+^ bound to TnC (states ‘B’ and ‘B^+^’ in Fig 1), and *i* RU’s where there is a TnI⋅TnC⋅Ca^2+^ state (‘S^+^’) in addition to the states with nothing or Ca^2+^ bound (states ‘U’ and ‘U^+^’). As in Fig 1, crossbridges can be bound in each of these states, although the term *E*_tm_ makes some of these combinations less probable (e.g. those corresponding to states ‘BX’ and ‘BX^+^’). Note that although states with detached TnI exist, where TnI is bound to neither TnC nor actin (‘U’ states in Fig 1), they are predicted by our model to be transient and unpopulated (at ∼ 1%) as both *K*_DI_ and *K*_DA_ are small.

**Table 1:** Index of model parameters.

Parameter	Description
$K_{\text{DA}}=e^{-\frac{E_{A}}{k_{\;B}T}}$	Dissociation constant for troponin I binding to actin
$K_{\text{DI}}=e^{-\frac{E_{I}}{k_{\;B}T}}$	Dissociation constant for troponin I binding to TnC⋅Ca^2+^
$K_{\text{DC}}=e^{-\frac{\left[{\text{Ca}}^{2+}\right]/E_{C}}{k_{\;B}T}}$	Dissociation constant for Ca^2+^ binding to TnC
$K_{\text{DM}}=e^{-\frac{E_{M}}{k_{\;B}T}}$	Dissociation constant for myosin binding to actin
*k*_A+_, *k*_A-_	On- and off-rate for troponin I binding to actin
*k*_I+_, *k*_I-_	On- and off-rate for troponin I binding to TnC⋅Ca^2+^
*k*_C+_, *k*_C-_	On- and off-rate for Ca^2+^ binding to TnC
*k*_M+_, *k*_M-_	On- and off-rate for myosin binding to actin
*γ*	Scaling parameter for tropomyosin properties

### Steady-state models

#### Steady-state model of thin filament kinetics

We start by developing a model which ignores crossbridge binding, and only calculates the number of unblocked RU’s. For this case *j* = 0 in Eq 4, and the tropomyosin state ‘tm’ is defined only by the points where TnI is bound to actin and moves tropomyosin to the ‘blocked’ position in each of the *n* = 26 RU’s, giving rise to 2^26^ ≈ 67 million states. We can further group the tropomyosin states from Eq 4 by their number of unblocked RU’s, while setting the number of crossbridges bound to *j* = 0. As only *E*_tm_ is dependent on the specific tropomyosin state, and the other terms are only dependent on the number of unblocked RU’s, this results in:

$$P\left(N_{u}\left(\text{tm}\right)=i\right)∼{\text{SE}}_{i}\;\frac{1}{K_{\text{DA}}^{n-i}}\;{\left(1+\frac{\left[{\text{Ca}}^{2+}\right]}{K_{\text{DC}}}\right)}^{n-i}\;{\left(1+\frac{\left[{\text{Ca}}^{2+}\right]}{K_{\text{DC}}K_{\text{DI}}}+\frac{\left[{\text{Ca}}^{2+}\right]}{K_{\text{DC}}}\right)}^{i}$$ (5)

$${\text{SE}}_{i}=\sum_{\left\{\text{tm}|N_{u}\left(\text{tm}\right)=i\right\}}e^{-\frac{E_{\text{tm}}}{k_{\;B}T}}$$ (6)

Thus, despite the large number of thin filament states, the *n* + 1 constants (SE_0_ to SE_*n*_) are sufficient to determine the relation between calcium concentration and (un)blocking of RU’s in the absence of crossbridges. Furthermore, these constants are sums of *E*_tm_ terms that can be readily determined from the flexible chain model of the thin filament. Although this process requires calculating 2^26^ finite element solutions to *E*_tm_, this is computationally tractable.

#### Steady-state model including crossbridges

The model described by Eq 5 does not yet include the effect of myosin crossbridges, which also displace the tropomyosin filament. Including these effects will be key in predicting the effects of XB-RU and XB-XB interactions. Crossbridges binding and unbinding to actin-tropomyosin are represented similarly to TnI binding to actin-tropomyosin, using a dissociation constant and an effect on tropomyosin deformation through the term *E*_tm_. For a full model which includes XB-XB and XB-RU interactions through the effects of both troponin I and crossbridges binding to actin on the deformation of the tropomyosin filament, we can use Eq 4 to determine the probability of being in a state with *i* unblocked RU’s and *j* crossbridges bound as:

$$P\left(N_{u}\left(\text{tm}\right)=i\wedge N_{\text{xb}}\left(\text{tm}\right)=j\right)∼\;{\text{SE}}_{i,j}\;\frac{1}{K_{\text{DA}}^{n-i}K_{\text{DM}}^{j}}\;{\left(1+\frac{\left[{\text{Ca}}^{2+}\right]}{K_{\text{DC}}}\right)}^{n-i}\;{\left(1+\frac{\left[{\text{Ca}}^{2+}\right]}{K_{\text{DC}}K_{\text{DI}}}+\frac{\left[{\text{Ca}}^{2+}\right]}{K_{\text{DC}}}\right)}^{i}$$ (7)

$${\text{SE}}_{i,j}=\sum_{\left\{\text{tm}\;|\;N_{u}\left(\text{tm}\right)=i,\;N_{\text{xb}}\left(\text{tm}\right)=j\right\}}e^{-\frac{E_{\text{tm}}}{k_{\;B}T}}$$ (8)

Eq 7 is similar to Eq 5, but now depends on both the number of unblocked RU’s and the number of crossbridges in a half-sarcomere. The SE_*i*,*j*_ values denote the sum of Boltzmann terms for all tropomyosin states with *i* RU’s unblocked and *j* crossbridges bound. In this initial investigation we do not consider details of sarcomere geometry and filament overlap effects, but simply model myosin binding sites as evenly spaced every 14.5 nm along the 1001 nm long filament [43, 49], resulting in *m* = 69 potential crossbridges per half-sarcomere. Including the displacement of tropomyosin introduced by these cross-bridges results in 2^69^ myosin states for each of the 2^26^ configurations of the thin filament RU’s. Unlike in the previous section, this large number of states makes the full computation of the state space computationally untraceable, and an approximation is required.

#### Sampling crossbridge states

To solve for the thin filament activation kinetics in the presence of XB’s requires evaluation of the 26 × 69 values SE_*i*,*j*_. Evaluating these values requires the solution of 2^26^ ⋅ 2^69^ tropomyosin states, which is not tractable. We combine two techniques for approximating these terms without requiring a brute-force calculation.

**Fig 2:**
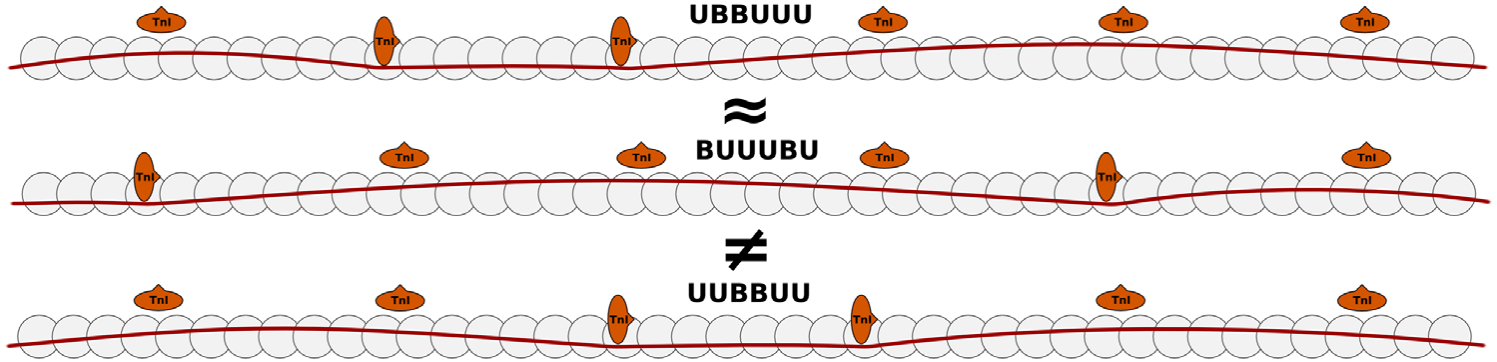
Equivalent tropomyosin states. The top two states are considered equivalent, and part of the same class of states, as they both have three adjacent unblocked RU’s and one isolated unblocked RU. The bottom state is part of a different class, even though it also has four unblocked RU’s, as it has two stretches with two adjacent unblocked RU’s each.

Firstly, in exploring smaller models we found that the crossbridge binding properties of the thin filament are dominated by the number and length of adjacent stretches of unblocked RU’s. Thus, if ‘B’ and ‘U’ indicate blocked and unblocked RU’s respectively, the crossbridge binding properties of the states ‘UBBUUU’ and ‘BUUUBU’ can be well approximated as identical. However, unlike some previous models [38], crossbridge binding properties can not be inferred from only the number of unblocked RU’s, such that (e.g.) the states ‘BUUUBU’ and ‘UUBBUU’ have significantly different crossbridge binding properties even though they both have four unblocked RU’s. Fig 2 illustrates these example states. This reduction results in 3010 *classes* of thin filament states which are equivalent in terms of crossbridge binding properties. We designate the state with the Boltzmann term $e^{-\frac{E_{\text{tm}}}{k_{\;B}T}}$ closest to the mean of the class as the ‘representative state’ for that class. For example, the ‘fully blocked’ and ‘fully unblocked’ states have their own class, the class with one unblocked RU represents 26 states (one for each position), and the class with two disconnected unblocked RU’s represents 300 states (each pair of disconnected positions). The standard deviation of the free energy within a class was on average 0.06% and in the worst case 0.11% of the free energy of the representative state of a class. This procedure allows us to perform subsequent calculations only on the 3010 representative states, instead of all 2^26^ thin filament states, but does not reduce the large number of crossbridge states, and still leaves us with 2^69^ cross-bridge states to be calculated for each of these representative classes. Additional results for the representative classes and states are shown in S2 Fig.

Secondly, for each of these representative classes, we approximate the sums of Boltzmann terms in Eq 8 by using a Monte Carlo approximation of the sum by random sampling. Technical details of this sampling procedure are described in S1 Text.

#### Independent crossbridge approximation

An alternative strategy for reducing the number of crossbridge states assumes that crossbridge binding does not significantly affect RU unblocking or binding of further crossbridges. Specifically, the model includes RU-RU cooperativity, and each crossbridge binding is affected by the current state of RU’s and corresponding tropomyosin deformation, but the resulting deformation of tropomyosin after crossbridge binding does not affect RU kinetics or other crossbridges. Creating a model which assumes XB-RU and XB-XB effects are negligible provides a baseline for comparing results of more detailed models and in determining the importance of XB-XB and XB-RU effects. Given this assumption, we can solve for the tropomyosin deformation without any crossbridges bound to get the free energy *E*_tm_, and with a single crossbridge bound to get $E_{\text{tm}}^{\prime }$ and the difference in tropomyosin free energy due to crossbridge binding $\Delta E_{\text{tm}}=E_{\text{tm}}^{\prime }-E_{\text{tm}}$. Using Boltzmann’s law, the *duty ratio* of a crossbridge, or the fraction of time it is expected to be bound to actin, is given by:

$$\frac{P(\text{xb}\;\text{on})}{P(\text{xb}\;\text{on})+P(\text{xb}\;\text{off})}=\frac{1}{1+K_{\text{DM}}\;e^{\frac{\Delta E_{\text{tm}}}{k_{\;B}T}}}$$ (9)

Where *P*(xb off), *P*(xb on) denote the probability of a specific crossbridge xb being off or on, and *K*_DM_ the dissociation constant for myosin as used previously. Tension generation in this simplified model is proportional to sum of duty ratios of all potential crossbridges for a representative tropomyosin state.

#### Parametrization of the steady-state model

The steady state model has five parameters: four dissociation constants and a parameter for the finite element model of the continuous flexible tropomyosin chain. In this section we determine these parameters for the full 26 RU model based on cooperative activation in intact muscle at body temperature, to reproduce general cooperative activation as observed across different species. Whenever possible we give parameters to a number of significant digits which reflects parameter sensitivity and experimental constraints.

We start by setting the dissociation constant of myosin *K*_DM_ based on the average duty ratio of crossbridges in a fully activated thin filament (determined by the average fraction of crossbridges bound), which is largely independent of other dissociation constants.

Estimates for the duty ratio of myosin vary from 5–10% for myosin heads in an actin-activated myosin ATPase assay [50], 14% in-vivo based on power-stroke distance [44], and up to approximately 30% at high force in experiments on human muscle fibers [51]. We set *K*_DM_ = 2, which results in a crossbridge duty ratio of 25% at full activation (1000 μM Ca^2+^), which is consistent with previous modelling work [38]. This value is at the higher end of experimental measurements but includes XB-XB interactions, and represents a myosin dimer rather than an isolated head.

Next, we determine the dissociation constants for competitive binding of TnI to TnC⋅Ca^2+^ (*K*_DI_) and actin (*K*_DA_) along with the scaling parameter *γ* of tropomyosin properties (bending stiffness and electrostatic interactions, see S1 Text). These parameters all influence cooperativity, while the remaining parameter *K*_DC_ only affects calcium sensitivity. Varying *K*_DI_ and *K*_DA_ between 10^−4^ and 0.1 shows the maximum Hill coefficient is *n*_*H*_ ≈ 4 when *γ* = 1, rising to *n*_*H*_ ≈ 6 for *γ* = 2 and *n*_*H*_ ≈ 8 for *γ* = 3. Based on measurements of the Hill coefficient below and above Ca_50_ (*n*_2_, *n*_1_ respectively), and the average Hill coefficient *n*_*H*_ required to replicate dynamic muscle function in phenomenological models [35, 36], we use *γ* = 2.

**Fig 3:**
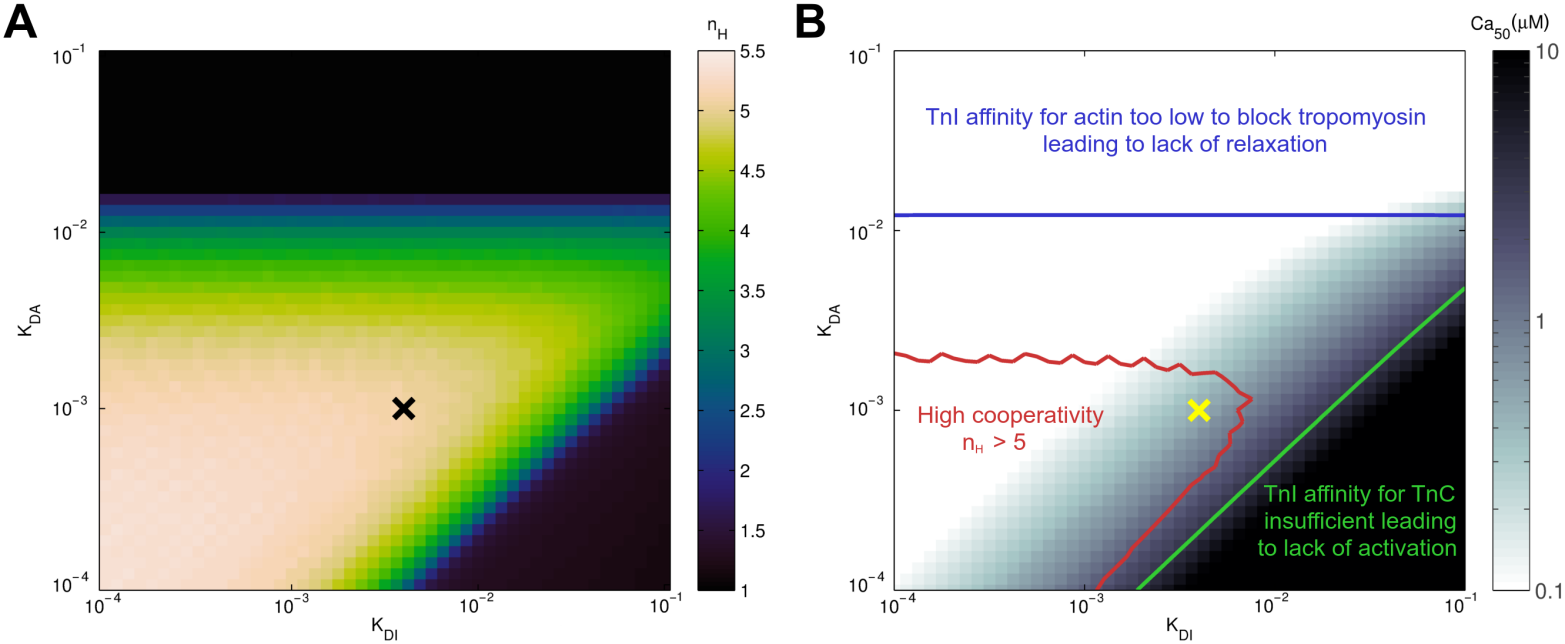
Influence of TnI affinity for actin and TnC on muscle cooperativity. Panel A shows cooperativity plotted as a function of the dissociation constant of TnI for actin (*K*_DA_) and the dissociation constant of TnI for TnC⋅Ca^2+^ (*K*_DI_). There is a relatively large triangular region in parameter space in which cooperativity is high, with a slight tendency for higher cooperativity at very low *K*_DA_, *K*_DI_ reflecting more extreme competitive binding of TnI. Panel B shows calcium sensitivity, which follows a smooth gradient The yellow ‘X’ indicates our choice of parameters, and the red contours indicate the regions within which *n*_*H*_ ≥ 5. At high *K*_DA_, affinity for actin is insufficient to block tropomyosin effectively, leading to a permanent high level of activation (indicated by the blue text and contour line for minimum force greater than 1% of maximum force at the top of the plot). When the affinity of TnI for actin is much lower than for TnC, muscle activation is decreased, (indicated by the green text and contour line for maximum force less than 95% of overall maximum force in the bottom right of the plot).

Fig 3 shows results for force-calcium relationships as a function of *K*_DI_ and *K*_DA_. This parameter sensitivity study shows that there is a relatively large triangular region in which cooperativity is high. We choose parameters sufficiently far away from regions where activation is impaired as indicated in Fig 3B, such that contractile function is maintained even if *K*_DA_, *K*_DI_ are varied (by e.g. phosphorylation of TnI). Additionally, given the lack of a clear lower bound, we consider very high affinities (i.e. very low *K*_DA_, *K*_DI_) to be physiologically less plausible due to larger differences in free energy between states. Within these constraints we choose *K*_DI_ = 4 ⋅ 10^−3^ and *K*_DA_ = 10^−3^, which results in high cooperativity (*n*_2_ = 7.5, *n*_1_ = 2.7) consistent with experimental data [52, 53],

Finally, we set *K*_DC_ = 5.9 μM based on a half-activation value Ca_50_ for the force-calcium relationship of approximately 0.5 μM, consistent with requirements of dynamic models with a peak calcium at the lower end of typical physiological range of 0.5–1 μM [54], experimental data (*K*_DC_ ≈ 5 μM [19]) and previous estimates of *K*_DC_ in models (between 1 and 10 μM [37]).

#### Testing approximation strategies for the crossbridge model

We proposed two strategies in making the model with crossbridges computationally tractable: the reduction of tropomyosin states to representative states based on connected stretches of unblocked RU’s (c.f. Fig 2), and subsequent Monte Carlo sampling with *n*_*s*_ = 1000 samples per representative state. In addition we proposed a much simpler ‘independent crossbridge model’ which does not include XB-RU and XB-XB interactions. To determine which of these models provides the best compromise between accuracy and computational tractability, we compare them with a brute-force approach on a smaller filament with 7 RU’s and 18 crossbridges. With 2^7+18^ ≈ 33 million calculations for the tropomyosin bending energy *E*_tm_, this is the largest thin filament for which a brute force approach is currently tractable.

**Fig 4:**
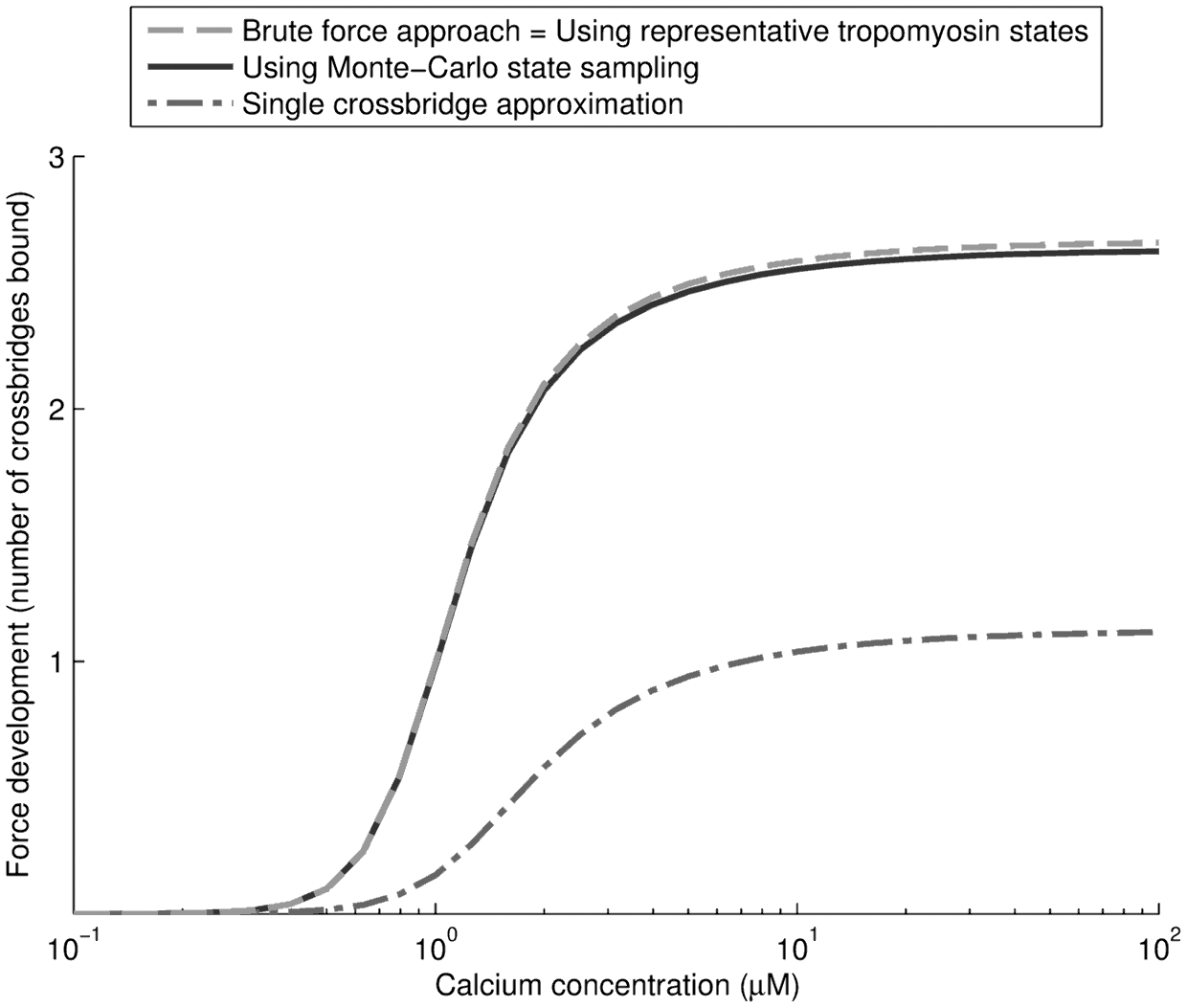
Comparison of different modelling approaches on a short filament. Shown are the different modelling approaches on a filament with *n* = 7 RU’s and 18 crossbridges. Results for the ‘brute-force solution’, and the representative state approximation overlap and are indicated by a single line. The Monte Carlo approximation performs well, with only a ∼ 1% difference at higher force levels. Comparison with the independent crossbridge approximation shows the importance of including XB-RU cooperativity, which increases calcium sensitivity, as well as XB-XB cooperativity, which increases maximum force development as shown by the difference in the number of crossbridges bound per half-sarcomere at high calcium. Note that due to the lower number of 7 RU’s, cooperativity is significantly lower than in realistic models with 26 RU’s presented in other results, and the duty ratio is moderately reduced to approximately 2.7/18 = 15%.

Results in Fig 4 are based on four simulation results: explicit calculation of all tropomyosin states, approximation of RU (un)blocking with representative states with with brute-force crossbridge calculation, further approximation of crossbridge states with Monte Carlo sampling, and an approximation with the assumption of independent crossbridge binding. Results indicate the ‘representative state’ approximation (with brute-force crossbridge calculation) overlaps completely with an exhaustive brute-force approach, i.e. the state of tropomyosin and its ability to bind myosin is very well approximated from the number and length of connected unblocked regulatory units. The Monte Carlo sampling approach also approximates the accurate solution well, with maximal differences of 1.2% at higher force levels. By contrast, the independent crossbridge approximation is shown to significantly underestimate force, by approximately 50%.

The difference between the independent crossbridge model and the brute force model can be attributed to two effects. Firstly, as nearly all RU’s are unblocked at the maximum calcium concentration, the difference in maximal force can be attributed to XB-XB cooperativity, i.e. the shifting of tropomyosin by a crossbridge makes it easier for neighbouring crossbridges to attach. Secondly, there is a significant shift towards lower calcium sensitivity in the independent crossbridge approximation, which is attributed to XB-RU cooperativity, where bound crossbridges inhibit the transition of tropomyosin to the ‘blocked’ state, effects which are also absent in the independent crossbridge approximation. All subsequent steady state analysis is performed on the representative state model with Monte Carlo crossbridge sampling.

#### Results for cooperative activation and XB-RU interactions

**Fig 5:**
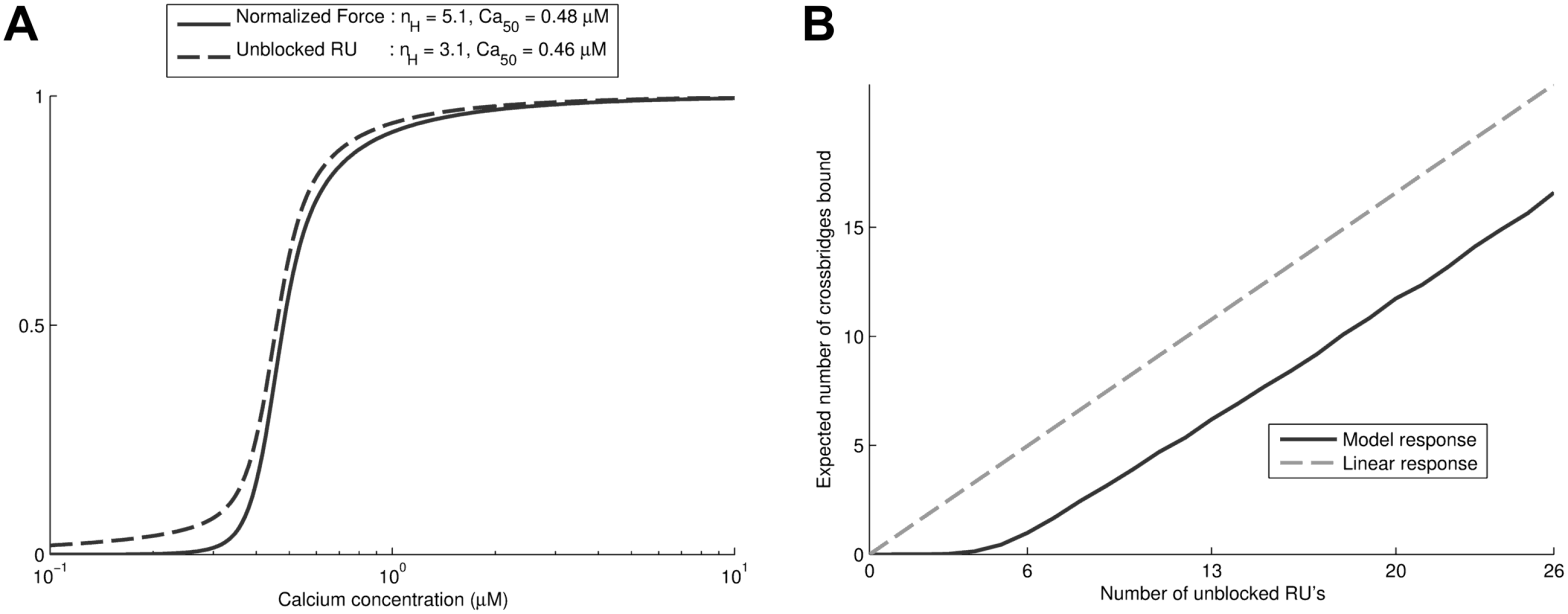
Steady-state model behaviour. Panel A shows the Force-calcium curve relationship of the model alongside the RU unblocking as a function of calcium. This shows that the Force-calcium curve is significantly steeper than RU activation, as indicated by its higher Hill coefficient. Panel B explains this effect by looking at the expected number of crossbridges on tropomyosin chains with a fixed number of RU’s unblocked, where the probability of tropomyosin sub-states is according to Boltzmann’s law. This shows that there is significant inhibition of crossbridge binding at low numbers of RU’s, compared to models where the ‘linear response’ of crossbridge binding being proportional to the number of unblocked RU’s is assumed.

In this section we use the full 26 RU model developed using the Monte Carlo sampling approach to investigate steady-state cooperative activation and XB-RU effects. Fig 5A shows cooperative tension development of the model, and compares it with the unblocking of RU’s. These results show that, at lower Ca^2+^, unblocking of RU’s is significantly less cooperative than force. This difference in cooperativity results in around 5% of RU’s still being unblocked at points when force is reduced to nearly zero. This difference is explained by low affinity of myosin for 1–4 neighbouring unblocked RU’s, shown in Fig 5B, and is a significant difference compared to most previous models which use a linearly increasing probability for crossbridge binding as a function of RU unblocking.

**Fig 6:**
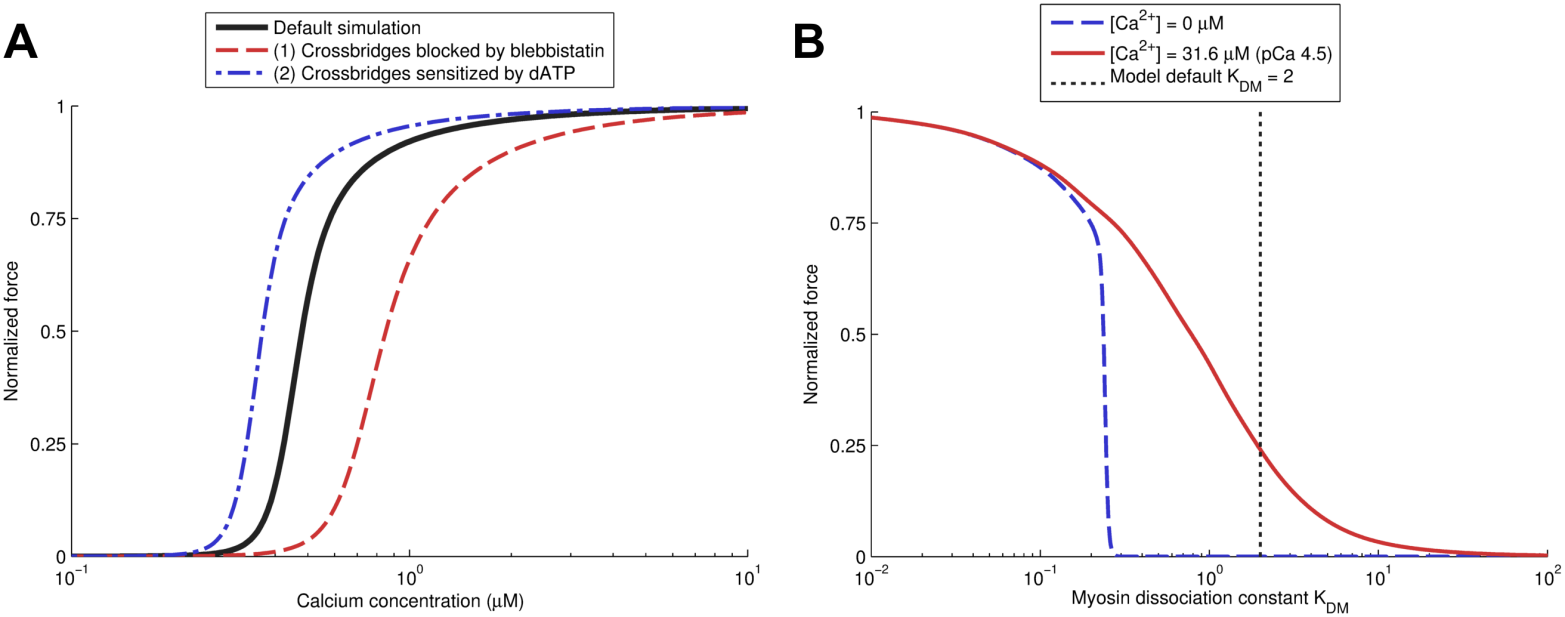
Force-pCa curves for the model and effects of changes in crossbridge affinity. The effects of crossbridge inhibition by substances such as blebbistatin and sodium vanadate were simulated by changing the dissociation constant of myosin (*K*_DM_), resulting in significant changes in calcium sensitivity. Shown in panel (A) are the default, highly cooperative, force-calcium relationship of the model (*n*_*H*_ = 5.1), along with the following virtual experiments: (1) In red: a factor 3 decrease in crossbridge affinity. This reproduces data from experiments with the cross-bridge inhibitor blebbistatin [16], showing a decrease in calcium sensitivity and a mild decrease in cooperativity (*n*_*H*_ = 4.2). (2) In blue: a 33% increase in crossbridge affinity. This reproduces data from experiments with the cross-bridge augmenter 2-deoxy-ATP (dATP) [55], showing an increase in calcium sensitivity and a small increase in cooperativity (*n*_*H*_ = 5.3). Panel (B) shows the *K*_DM_-dependence of force at zero Ca^2+^ and at pCa 4.5 [45], showing the model produces maximal force at both calcium levels for a sufficiently high myosin affinity, and a sigmoidal relationship between *K*_DM_ and force. The dashed line indicates the value of *K*_DM_ used in the model, which intersects the pCa 4.5 curve at approximately 0.25, the duty ratio of myosin used in the model. Thus, the maximal force generated in panel (B) for *K*_DM_ → 0 is approximately 4× higher than the ‘default model’ curve in panel (A).

Fig 4 presented in the previous section showed a shift in calcium sensitivity when XB-XB and XB-RU effects are included. There are several experiments which reveal important interactions between crossbridge affinity and calcium sensitivity, including experiments in which blebbistatin or sodium vanadate are used to decrease crossbridge affinity [1, 16, 54], and experiments with dATP where crossbridge affinity is increased [55]. In general, an increase in crossbridge affinity leads to higher calcium sensitivity, i.e. the muscle activating at lower [Ca^2+^]. We modeled the effects of blebbistatin by a decrease in myosin head affinity (3× higher *K*_DM_), and the effects of dATP by a higher myosin affinity (25% decrease in *K*_DM_). Increasing myosin affinity led to a leftward shift of the force-calcium curve, and vice versa, as shown in Fig 6A. The changes in crossbridge affinity for blebbistatin and dATP were fitted to the change in maximum force shown in experiments, resulting in good quantitative agreement for the predicted shift in calcium sensitivity (ΔpCa_50_), as indicated in Table 2. In addition, we tested the models ability to activate due a high myosin affinity as observed in conditions of low ATP [45]. For the rigor test we vary *K*_DM_ and record the force generation predicted by the model. The results (Fig 6B) are qualitatively similar to experimental data for pCa 4.5 [45], with a decreasing sigmoidal relationship. Thus, our model replicates the shifts in calcium sensitivity shown in experiments where crossbridge affinity is modified, and is also able to activate in the absence of significant calcium due to rigor crossbridges.

**Table 2:** Model predictions for shifts in pCa_50_ after changes in crossbridge affinity.

Experiment	Blebbistatin [16]	dATP [55]
Experimental conditions	Skinned, room temp., mouse	Skinned, 15°C, rat
Experimental max. force	-73%	+31%
Experimental ΔpCa_50_	-0.34	+0.13
Model conditions	Both models represent intact muscle, 37°C, mouse	
Model max. force (fitted)	-73.6%	+30.9%
Model ΔpCa_50_	-0.248	+0.117

Predictions of changes in calcium sensitivity (ΔpCa_50_) when fitting crossbridge affinity in the model to reproduce changes in maximal force shown experimentally. See Fig 6 for full traces.

### Dynamic model

The steady-state models developed in the previous sections replicate a range of experimental measurements related to steady-state cooperative activation. Cooperative effects also have an important impact on beat-to-beat dynamic tension generation, and may have different roles in different species due to differences in heart rate and calcium dynamics. To be able to investigate the role of cooperative activation in dynamic tension generation, in this section we extend our proposed framework to simulate dynamic changes in tension in response to transient changes in Ca^2+^.

For a dynamic model of *n* RU’s and *m* crossbridges, we use:

- A regular grid of (*n* + 1) ⋅ (*m* + 1) state variables TmXB_i,j_ which represent the fraction of half sarcomeres with *i* RU’s unblocked and *j* crossbridges bound.
- The state variable TnC_B_, the fraction of RU’s that are blocked with Ca^2+^ bound to TnC
- The state variable TnC_U_, the fraction of RU’s that are unblocked with Ca^2+^ (but not TnI) bound to TnC,
- The state variable TnITnC, the fraction of RU’s that are blocked with Ca^2+^ and TnI bound to TnC

Several dependent variables are useful in the formulation in the differential equations for these states:

$$U=\sum_{i=0}^{n}\sum_{j=0}^{m}\;\frac{i}{n}{\text{TmXB}}_{\text{i,j}}\;(\text{fraction of RU}’\text{s in the unblocked state})$$ (10)

B=1-U(fraction of RU’s in the blocked state) (11)

TnI=U-TnITnC(fraction of RU’s with TnI not bound to actin or TnC) (12)

The kinetics of the tropomyosin and crossbridge states can now be defined using a standard Markov model approach, with transition rates defined using the ratio of Boltzmann terms:

$$\begin{array}{cc} \frac{{\text{dTmXB}}_{\text{i,j}}}{\text{d}t} & =\overset{\text{if}\;i\neq 0}{\overset{︷}{k_{\text{i-1,j}}^{\text{b}\to \text{u}}{\text{TmXB}}_{\text{i-1,j}}-k_{\text{i,j}}^{\text{u}\;\to \;\text{b}}{\text{TmXB}}_{\text{i,j}}}}\;+\;\overset{\text{if}\;i\neq n}{\overset{︷}{k_{\text{i+1,j}}^{\text{u}\;\to \;\text{b}}{\text{TmXB}}_{\text{i+1,j}}-k_{\text{i,j}}^{\text{b}\to \text{u}}{\text{TmXB}}_{\text{i,j}}}}\\ & +\;\overset{\text{if}\;j\neq 0}{\overset{︷}{k_{\text{i,j-1}}^{\text{xb+}}{\text{TmXB}}_{\text{i,j-1}}-k_{\text{i,j}}^{\text{xb-}}{\text{TmXB}}_{\text{i,j}}}}\;+\;\overset{\text{if}\;j\neq m}{\overset{︷}{k_{\text{i,j+1}}^{\text{xb-}}{\text{TmXB}}_{\text{i,j+1}}-k_{\text{i,j}}^{\text{xb+}}{\text{TmXB}}_{\text{i,j}}}} \end{array}$$ (13)

$$k_{\text{i,j}}^{\text{xb+}}/k_{\text{i,j+1}}^{\text{xb-}}=\frac{{\text{SE}}_{i,j+1}}{{\text{SE}}_{i,j}}\cdot \frac{1}{K_{\text{DM}}}$$ (14)

$$k_{\text{i,j}}^{\text{b}\to \text{u}}/k_{\text{i+1,j}}^{\text{u}\to \text{b}}=\frac{{\text{SE}}_{i+1,j}}{{\text{SE}}_{i,j}}\cdot K_{\text{DA}}\cdot \frac{U}{\text{TnI}}$$ (15)

Where $k_{\text{i,j}}^{\text{u}\to \text{b}},k_{\text{i,j}}^{\text{b}\to \text{u}}$ are the transition rates from unblocked to blocked, and blocked to unblocked, respectively, for the state with *i* unblocked RU’s. The ratio $k_{\text{i,j}}^{\text{b}\to \text{u}}/k_{\text{i+1,j}}^{\text{u}\to \text{b}}$ follows naturally from the ratio of Boltzmann terms between the states, requiring only the addition of the probability that TnI is free to bind to actin *P*(TnI free∣RU unblocked) = TnI/*U*.

The framework based on Boltzmann’s law can be used to determine the ratios of transition rates, but does not result in an absolute on- and off-rate. To determine how the energy difference influences the on- and off-rates, we use a similar approach as proposed by Campbell et al. [38]. Given two states *S*_1_, *S*_2_ with energies *E*_1_, *E*_2_ and Boltzmann terms $B_{1}=e^{-\frac{E_{1}}{k_{\;B}T}},B_{2}=e^{-\frac{E_{2}}{k_{\;B}T}}$, the transition rates between them are given by:

$$k_{S_{1}\to S_{2}}/k_{S_{2}\to S_{1}}=B_{2}/B_{1}$$ (16)

$$k_{S_{1}\to S_{2}}={\left(B_{2}/B_{1}\right)}^{r}$$ (17)

$$k_{S_{2}\to S_{1}}={\left(B_{2}/B_{1}\right)}^{-(1-r)}$$ (18)

The parameter *r* represents how strongly the on-rate and off-rate depend on the difference in energy between the states *E*_1_/*E*_2_, ranging from only the off-rate (*r* = 0) to only the on-rate (*r* = 1). We apply this model to the effect of tropomyosin deformation on the rates $k_{\text{i,j}}^{\text{b}\to \text{u}},k_{\text{i,j}}^{\text{u}\to \text{b}}$, with the assumption that both rates are equally affected (*r* = 0.5). The affinity of TnI for actin *K*_DA_ is handled separately from the influence of tropomyosin, and is split into two rate constants *k*_A-_, *k*_A+_. In addition, as our state TmXB_i,j_ is a combination of several sub-states with *i* unblocked RU’s, what remains is to take into account is the difference in the number of transitions in the average state, given by (*n* − *i*) potential RU’s for a blocked-to-unblocked transition and *i* potential RU’s for a unblocked-to-blocked transition, for any state. Combined, these considerations result in the following equations for the rate constants:

$$k_{\text{i,j}}^{\text{b}\to \text{u}}=k_{\text{A-}}\cdot \left(n-i\right)\cdot {\left(\frac{{\text{SE}}_{i+1,j}/\left(n-i\right)}{{\text{SE}}_{i,j}/\left(i+1\right)}\right)}^{r}$$ (19)

$$k_{\text{i,j}}^{\text{u}\to \text{b}}=k_{\text{A+}}\cdot i\cdot {\left(\frac{{\text{SE}}_{i,j}/\left(n-i+1\right)}{{\text{SE}}_{i-1,j}/i}\right)}^{-\left(1-r\right)}\cdot \frac{\text{TnI}}{U}$$ (20)

For crossbridge binding and unbinding rates we introduce a parameter *q* to represent the effect of tropomyosin deformation on the crossbridge binding and unbinding rate. Two different choices will be considered. Firstly, tropomyosin deformation affecting both on- and off-rate equally (*q* = 0.5) similar to RU unblocking. Secondly, the choice of a constant unbinding rate (*q* = 1), where only the on-rate is affected by tropomyosin deformation. The impact of this choice will be considered in the next section. Taking into account the number of potential crossbridges, equations for the transition rates are given by:

$$k_{\text{i,j}}^{\text{xb+}}=k_{\text{M+}}\cdot \left(m-j\right)\cdot {\left(\frac{{\text{SE}}_{i,j+1}/\left(m-j\right)}{{\text{SE}}_{i,j}/\left(j+1\right)}\right)}^{q}$$ (21)

$$k_{\text{i,j}}^{\text{xb-}}=k_{\text{M-}}\cdot j\cdot {\left(\frac{{\text{SE}}_{i,j}/\left(m-j+1\right)}{{\text{SE}}_{j-1,i}/j}\right)}^{-\left(1-q\right)}$$ (22)

We model TnC and TnI kinetics using simplified global state variables for the blocked and unblocked regulatory units. The equations for these kinetics are given by:

$$\frac{\text{dTnITnC}}{\text{d}t}=k_{\text{I+}}{\text{TnC}}_{\text{U}}-k_{\text{I-}}\text{TnITnC}$$ (23)

$$\frac{{\text{dTnC}}_{\text{B}}}{\text{d}t}=k_{\text{C+}}\left[{\text{Ca}}^{2+}\right]\;\left(B-{\text{TnC}}_{\text{B}}\right)-k_{\text{C-}}{\text{TnC}}_{\text{B}}-J_{\text{bu}}+J_{\text{ub}}$$ (24)

$$\frac{{\text{dTnC}}_{\text{U}}}{\text{d}t}=k_{\text{C+}}\left[{\text{Ca}}^{2+}\right]\;\left(U-{\text{TnC}}_{\text{U}}-\text{TnITnC}\right)-k_{\text{C-}}{\text{TnC}}_{\text{U}}-\frac{\text{dTnITnC}}{\text{d}t}+J_{\text{bu}}-J_{\text{ub}}$$ (25)

These equations represent standard Michaelis-Menten kinetics, apart from the terms *J*_bu_ and *J*_ub_, which represent the ‘flux’ of Ca^2+^ bound to TnC between the global TnC buffers for blocked and unblocked RU’s. Each time an RU blocks or unblocks, we need to consider the probability of a calcium ion moving between TnC_B_ and TnC_U_. These fluxes are given by:

$$J_{\text{bu}}=\frac{1}{n}\frac{{\text{TnC}}_{\text{B}}}{B}\sum_{j=0}^{m}\sum_{i=0}^{n-1}k_{\text{i,j}}^{\text{b}\to \text{u}}{\text{TmXB}}_{\text{i,j}}$$ (26)

$$J_{\text{ub}}=\frac{1}{n}\frac{{\text{TnC}}_{\text{U}}}{\text{TnI}}\sum_{j=0}^{m}\sum_{i=1}^{n}k_{\text{i,j}}^{\text{u}\to \text{b}}{\text{TmXB}}_{\text{i,j}}$$ (27)

The sums represent the total transition rates between blocked and unblocked RU’s from Eq 13, multiplied by 1/*n* to represent one RU out of *n* changing for each transition. This is multiplied by the probability of a calcium ion being present on a closing or blocking RU, which is $\frac{{\text{TnC}}_{\text{B}}}{B}$ for blocked units and $\frac{{\text{TnC}}_{\text{U}}}{\text{TnI}}$ for unblocked units as this latter probability needs to be considered over unblocked RU’s which do not have TnI bound to TnC·Ca^2+^ (and $k_{\text{i,j}}^{\text{u}\to \text{b}}$ already contains a factor TnI/*U*).

For a numerical implementation, quantities such as TnI/*U* (in Eq 20) should be calculated as TnI/ max(*U*, *ɛ*) for a small constant *ɛ* to avoid undefined 0/0 quantities. Secondly, many of the states TmXB_i,j_ are not populated in practice and can be removed from the formulation for improved efficiency and stability. Specifically, for every *i* we include states TmXB_i,j_ up to the value of *j* for which SE_*i*,*j*_ < 10^−6^∑_*i*_ SE_*i*,*j*_, which reduces the number of states from 1794 to ∼ 750 without significantly affecting the solution. The model’s initial condition should be determined by pacing for a specific calcium transient and parametrization, starting from the completely de-activated state (all state variables set to 0 except TmXB_0,0_ = 1).

## Results

### Tension development in different species

In a physiological setting, cooperativity is a key component of normal activation and relaxation of the heart. Thus, an important test of the effectiveness of a model in reproducing physiological cooperativity is its ability to reproduce realistic tension based on experimentally measured calcium transients. In this section, we investigate if tension development across different species is consistent with identical cooperativity, despite significant differences in heart rate. Although the equations in the dynamic model appear to have a large number of free parameters, all transition rates follow from the 9 parameters listed in Table 3. We first parametrize our model to reproduce twitch tension at 37°C in mouse, as we have found this the most challenging test case in practice, and start with the *q* = 0.5 case. Active tension was calculated by setting the maximal tension developed to 120 kPa as in previous work [36] (see S1 Text for details). We vary *k*_A-_, *k*_I-_, *k*_M-_, *k*_C+_ between 0.01/ms and 100/ms, and found that tension development and relaxation are all sensitive to the choice of these parameters. As *k*_A-_, *k*_I-_ are generally not thought to be rate-limiting [56], we set both of them to 10/ms. Parameters *k*_M-_, *k*_C+_ are then determined by requirements for time to peak tension and relaxation times according to the ranges of experimental measurements determined in previous work [36] and summarized in Table 4, resulting in *k*_C+_ = *k*_M-_ = 0.5/ms.

**Table 3:** List of model parameters.

Parameter	Value	Determined based on
*K*_DA_	10^−3^	Cooperativity, effective competitive binding
*K*_DI_	4 ⋅ 10^−3^	Cooperativity, effective competitive binding
*K*_DC_	5.9 μM	Required Ca_50_, literature data, previous modelling [19, 37]
*K*_DM_	2	Duty ratio in fully activated muscle
*k*_A-_	10/ms	High enough to not be rate-limiting
*k*_I-_	10/ms	High enough to not be rate-limiting
*k*_C+_	0.5/ms	Fitting to TPT, RT, Maximum twitch force
*k*_M-_	0.5/ms	Fitting to TPT, RT, Maximum twitch force
*γ*	2	Cooperativity *n*_2_ ≈ 8

Shown here are all model parameters for the mouse model with the method used for parametrization.

**Table 4:** Parametrization of the model for different species.

	Mouse	Rat	Human
Parametrization with variable XB unbinding rates (*q* = 0.5)			
*K*_DI_	4 ⋅ 10^−3^	8.7 ⋅ 10^−3^	2.6 ⋅ 10^−3^
*k*_M-_ (ms^−1^)	0.5	0.09	0.016
Model TPT (ms)	33	41	170
Model RT_50_ (ms)	29	30	124
Model RT_90_ (ms)	49	51	217
Model RT_95_ (ms)	56	57	247
Parametrization with constant XB unbinding rates (*q* = 1)			
*K*_DI_	4 ⋅ 10^−3^	8.7 ⋅ 10^−3^	3.3 ⋅ 10^−3^
*k*_M-_ (ms^−1^)	2	0.3	0.049
Model TPT (ms)	33	37	157
Model RT_50_ (ms)	28	29	125
Model RT_90_ (ms)	49	55	250
Model RT_95_ (ms)	57	64	298
Experimental data			
TPT (ms)	30–40	39 ± 6	147–172
RT_50_ (ms)	22–35	31 ± 5	109–125
RT_90_ (ms)	45–60		
RT_95_ (ms)			334 ± 43
References	[57–61]	[62]	[63, 64]

**Fig 7:**
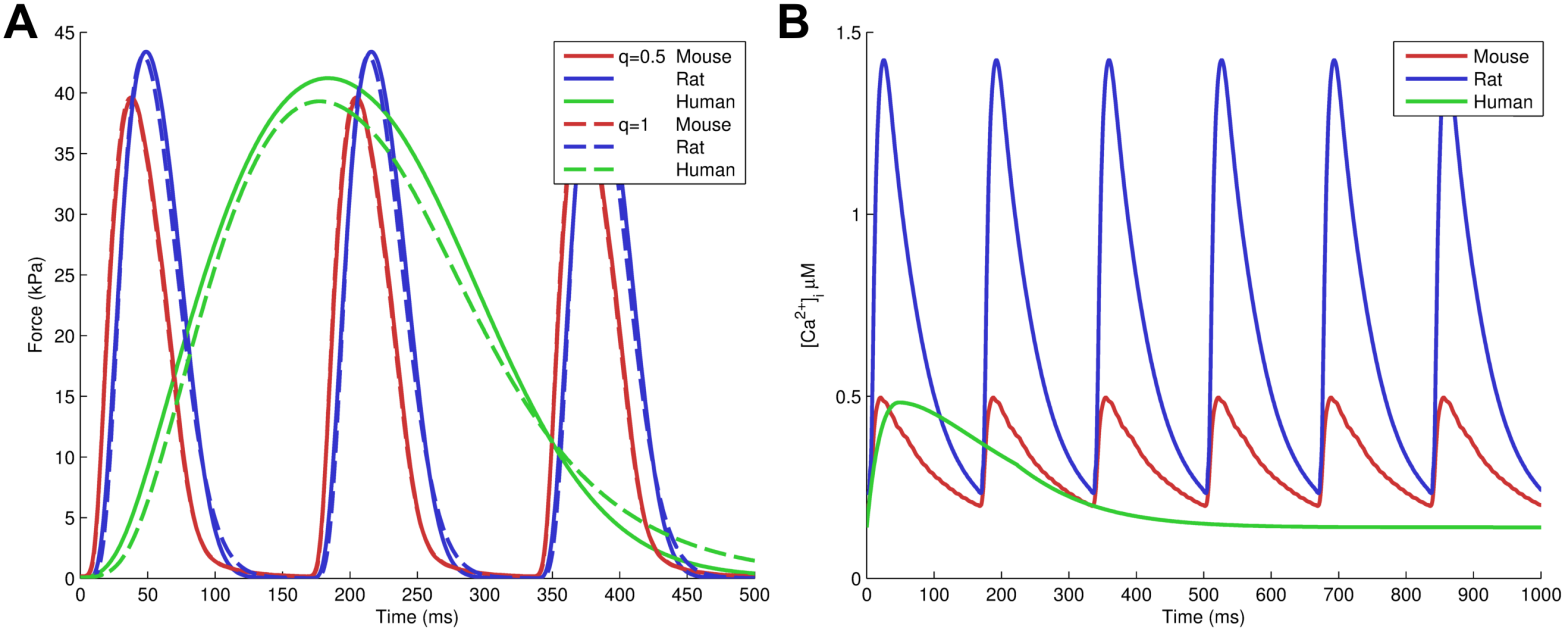
Isometric tension in mouse, rat, and human. Shown in panel A are the tension transients described in Table 4. The last 500 ms of the human tension transients are not shown, as force is very low throughout. These results were driven by fixed calcium transients shown in panel B, based on recent data in mouse [66] and rat (see S1 Text [67, 68]) measured at 37°C and 6 Hz, and data from Coppini et al. [69] (see S1 Text [70]). Two sets of results are shown, corresponding to a constant crossbridge unbinding rate (*q* = 1 in Eq 22) and a crossbridge unbinding rate that is variable, modified by the average difference in free energy for the tropomyosin deformation (*q* = 0.5 in Eq 22)

Shown in this table are the changes to *K*_DI_, *k*_M-_ needed to replicate mouse, rat and human dynamic muscle function. Also shown are metrics of time to peak tension (TPT) and 50%/90%/95% relaxation times (RT_50_, RT_90_, RT_95_) of the dynamic model. Results for force transient are also shown in Fig 7. The bottom section shows experimental data ranges used for parametrization of the models. Two sets of results are shown, corresponding to a constant crossbridge unbinding rate (*q* = 1 in Eq 22) and a crossbridge unbinding rate that is variable, modified by the average difference in free energy for the tropomyosin deformation (*q* = 0.5 in Eq 22)

Based on this initial parametrization, we investigate dynamic function of the model across species, applying it to both tension generation in isometric twitches and tension redevelopment. Firstly, we parameterize the model for three different species as well as both choices of crossbridge binding rates (*q* = 1 or *q* = 0.5). Troponin C is a highly conserved protein and its kinetics are not sensitive to temperature or (mammalian) species [56] while crossbridge cycling rates are highly dependent on species, temperature and myosin heavy chain isoform. However, despite the lack of variation in TnC properties, calcium transients vary between the different species while a similar peak isometric tension of approximately 40 kPa is required to be consistent with whole organ contraction across different species [36, 70]. Inspired by these observations we use our model to test the hypothesis that contraction across different mammalian species (mouse, rat and human) can be reproduced using only differences in TnI-TnC affinity as given by *K*_DI_ and the rate of crossbridge kinetics as given by *k*_M-_. We determined these two parameters by using a two-dimensional parameter sweep which shows the influence of different constraints on both parameters. Constraints used include species-specific constraints for time to peak tension and relaxation times, and constraints on minimum force < 1 kPa and maximum force between 35–45 kPa in all species. Table 4 shows the parametrization for the three different species [57–64], and Fig 7 shows the corresponding force transients. We were able to capture the different twitch kinetics between species with variations in only *K*_DI_ and *k*_M-_. The resulting parameters show that changes in crossbridge kinetics are consistent with differences in heart rate (mouse > rat > human), while changes to the parameter *K*_DI_ for TnI affinity for TnC⋅Ca^2+^ correspond to differences in the calcium transients (c.f. Fig 7B). With respect to the choice of crossbridge unbinding rate on the thin filament state (*q* = 1 or *q* = 0.5), the parameter *K*_DI_ could be kept the same for the different choices although it was not fixed a priori. However, *k*_M-_ needs to be significantly higher when constant unbinding rates are used. Overall, model results suggest contractile function across these different species are consistent with a common mechanism and kinetics for thin filament cooperative activation.

### Simulating tension redevelopment

The rate of tension redevelopment (*k*_tr_) has been shown to vary significantly depending on [Ca^2+^], with experiments showing a range of 4–8× between the lowest and highest rates observed [55, 71–74]. These differences in tension redevelopment rates have been linked to the effect of thin filament activation kinetics [55], and is often not well reproduced by computational models. To test our model’s ability to replicate and explain these more complex dynamic effects which involve multiple contractile proteins, and isolate the role of cooperativity in tension redevelopment, we have performed simulations of the calcium dependence of the redevelopment rate of tension.

As our model does not account for dynamic length changes, we apply the following procedure to simulate the *k*_tr_ protocol. For each calcium level the model is run to steady-state, and 50% of crossbridges are instantly detached to simulate the state of the filament after a rapid shorten-relengthen protocol (c.f. trace in [71]). Crossbridges are detached by setting TmXB_i,j/2_ to TmXB_i,j_ for even *j*, and TmXB_i,⌈j/2⌉_ and TmXB_i,⌊j/2⌋_ to $\frac{{\text{TmXB}}_{\text{i,j}}}{2}$ for odd *j*, where ⌈⋅⌉, ⌊⋅⌋ denote rounding up and down, respectively. Subsequently the model is run with normal binding rates to simulate tension redevelopment, and mono-exponential curve is fitted to the resulting force:

$$\frac{F}{F_{\text{max}}}=1-ae^{-k_{\text{tr}}t}$$ (28)

**Fig 8:**
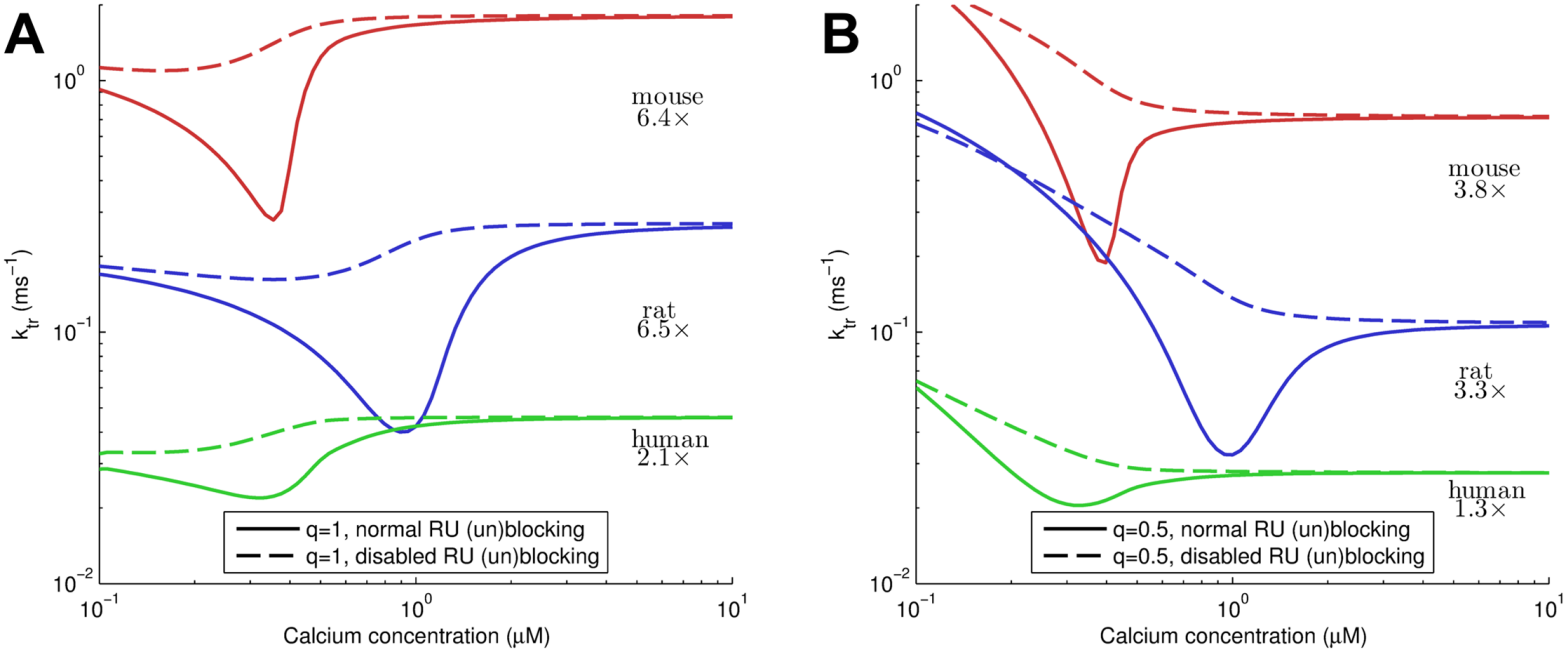
Tension redevelopment rates in mouse, rat and human. Panel A shows results for *q* = 1 (constant crossbridge unbinding rate), and Panel B plot results for *q* = 0.5 (variable crossbridge unbinding rate). Each plot shows both normal kinetics, and with RU (un)blocking due to TnI-actin binding disabled for mouse, rat, and human parametrizations of the model. The difference in *k*_tr_ when disabling RU (un)blocking reveals the influence of transient blocking of RU’s on tension redevelopment kinetics. This transient blocking causes a significant difference between the minimum *k*_tr_ and the *k*_tr_ at high [Ca^2+^], the ratio of which is indicated with the results. Note the shift in the minimum *k*_tr_ is not significant here as it is a direct result of the difference in peak calcium shown in Fig 7A and subsequent parametrization.

To investigate the importance of XB-RU cooperative activation, this procedure is repeated with disabled TnI-actin dynamics (*k*_A-_ = *k*_A+_ = 0) during tension redevelopment to prevent RU (un)blocking. Results in Fig 8 show that a constant unbinding rate independent of tropomyosin deformation (*q* = 1) shows a larger difference between the minimal *k*_tr_ and the value at high Ca^2+^, with around a 6.5× difference in mouse and rat. Interestingly, unlike experimental data, our results show a clear minimum rather than the typical monotonically increasing *k*_tr_. We attribute this effect to the smaller range of [Ca^2+^] investigated in experiments and the tendency for noise to dominate measurements at low force levels. For a variable unbinding rate, this ratio is only around 3–4 in mouse and rat. In addition, *k*_tr_ rises steeply at low [Ca^2+^], as the unbinding rate becomes very fast. In both cases, transient blocking of RU’s is the main mechanism leading to the minimum in *k*_tr_. At very low and high [Ca^2+^], the state of the thin filament is not much affected by crossbridges, being mostly blocked or unblocked regardless. This can be seen by the similar *k*_tr_ for results with and without RU (un)blocking disabled. However, at intermediate [Ca^2+^], unblocking of crossbridges will cause RU’s to start moving to the ‘blocked’ position, requiring additional time to be re-activated by XB-RU interaction resulting in the lower *k*_tr_.

## Discussion

In this paper we developed a novel model of cardiac contraction and used it to investigate the effects of different cooperative mechanisms on both the steady-state and dynamic behaviour of cardiac muscle. We have been able to show the relative importance of the different potential mechanisms for generating the steeply cooperative force-calcium relationship in muscle. Firstly, RU-RU cooperativity is the clear dominant mechanism for cooperativity near and above Ca_50_, caused by progressively easier unblocking for any individual RU as more of their close neighbours are unblocked. This can be seen from the cooperativity of the RU unblocking in the absence of crossbridges (Fig 4) and RU activation (Fig 5), with moderate cooperativity which is not significantly biphasic. Although RU-RU cooperativity deriving from end-to-end interactions between tropomyosin is a major part of cooperative activation, it does not explain all of the cooperative activation seen.

Secondly, cooperativity between crossbridges, i.e. of the ‘XB-XB’ type, approximately doubles the number of crossbridges bound at maximal activation (Fig 4), and in doing so increases the effects of XB-RU interactions, but does not in itself significantly increase the steepness of the force-calcium relationship. Thirdly, we have shown that activation of regulatory units by crossbridges, i.e. ‘XB-RU’ cooperativity, is important for determining calcium sensitivity. Our model reproduces the effects of changes in the affinity of myosin XB on calcium sensitivity shown experimentally [1, 16, 54] (Fig 6). Despite differences in experimental conditions due to temperature and permeabilization of muscle, there is good quantitative agreement in the shift in calcium sensitivity (ΔpCa_50_, Table 2). Our results show a mild decrease in cooperativity for simulations with decreased crossbridge affinity (*n*_*H*_ = 5.1 → 4.2) which is almost entirely in the lower half (below Ca_50_) of the force-calcium relationship (*n*_2_ = 7.5 → 6.1), with nearly identical values for the upper half (*n*_1_ = 2.7 → 2.4). The modest change in Hill coefficient may explain some of the contradictory results in the literature so far, as Hill fits are inherently sensitive to the choice of fitting method and calcium concentrations used, in addition to experimental noise. The ratio of *n*_2_/*n*_1_ varies between 2.5–2.7 in these simulations, compared to approximately 2.0 in experiments [52, 53]. This ratio is very sensitive to the choice of fitting methods and [Ca^2+^] window used for fitting, which could explain these differences. Alternatively, these differences could be the result of the simplified sarcomere geometry in the current model. In addition, our model reproduces the activation of muscle by high-affinity ‘rigor’ crossbridges, such as in conditions of low ATP [45] or special experimental preparations with NEM-S1 myosin [75], even in the complete absence of Ca^2+^. Although results from these experiments are less important for contraction models to reproduce as they represent conditions far from physiological, they are a direct result of XB-RU cooperativity in our model and thus increases confidence in the choice of biophysical framework. In addition this is a novel feature for computational models, as in the vast majority of models in this area RU’s are mathematically unable to activate in the absence of Ca^2+^ [34–36, 38]. Our model also explains effects of force on Ca^2+^-TnC binding. The proposed effects of crossbridges on Ca^2+^-TnC affinity appear in our model as an emergent property of the competitive binding of TnI and pinning of tropomyosin by both TnI and crossbridges. Specifically, crossbridge binding prevents tropomyosin to move to the blocked position, and this effectively prevents TnI from binding to tropomyosin-actin. This in turn increases the relative time TnI is bound to TnC⋅Ca^2+^, thus increasing the effective affinity of TnC for Ca^2+^, as Ca^2+^ is highly unlikely to dissociate from TnC when TnI is bound. As a result, our framework represents the observed tension-dependent feedback on TnC affinity without hysteresis in the steady-state force-pCa relationship.

Finally, we have identified an important effect by which approximately five neighbouring unblocked tropomyosin units are required to significantly bind myosin (Fig 5). These effects are also partly responsible for the very steep force-calcium relationship in the region below Ca_50_ and the strongly biphasic shape of the force-calcium curve. Specifically, it allows for near-zero force in a state with a significant fraction of unblocked RU’s with Ca^2+^ bound to TnC, as there are not enough consecutive unblocked RU’s to generate significant force. Achieving low force at physiological diastolic [Ca^2+^] of approximately 0.1–0.2 μM is particularly important for effective diastolic filling in the heart. In addition this result shows that the common modelling assumptions of crossbridge binding properties being linearly proportional to the number of unblocked RU’s is potentially inaccurate.

Thus, cooperative activation of muscle derives in part from the end-to-end interactions of tropomyosin, with additional cooperativity mostly below Ca_50_ driven by XB-RU effects and nonlinear crossbridge binding properties. Building on the steady-state models, we developed different models for dynamic muscle function in mouse, rat and human, to investigate the ability of our model of cooperative activation in reproducing physiological activation and relaxation of muscle. We have parameterized the model to these three different species using only changes in the affinity of TnI for TnC (*K*_DI_) and crossbridge cycling rates (*k*_M-_). Our results in Table 4 and Fig 7 show it is possible to reproduce the different kinetics and accommodate large changes in Ca^2+^ transients without changing the properties of TnC which are generally thought to be highly conserved. Our results are consistent with a highly conserved underlying mechanism for cooperative activation, while the relative order of crossbridge cycling rates is highly species dependent as expected from the differences in heart rates, with mouse faster than rat, and rat faster than human.

The model also naturally reproduces the Ca^2+^-dependence of the rate of force redevelopment *k*_tr_, and is able to explain the strong Ca^2+^ dependence of force redevelopment as a result of transient blocking of RU’s (Fig 8). Specifically, detachment of crossbridges causes a steady-state activation that is lower (similar to the blebbistatin experiments in the steady-state models), and results in blocking of RU’s due to a decrease in XB-RU effects. At high and low Ca^2+^, the filament state is mostly blocked or unblocked, and is not much affected by the number of crossbridges. However, near Ca_50_, this state is particularly sensitive to crossbridges and XB-RU effects. As crossbridges are detached by the fast length change, some RU’s move to a blocked position, and require additional time to become unblocked again through XB-RU cooperative interactions. This causes a higher *k*_tr_ compared to that seen at high Ca^2+^ concentrations. The choice of constant or tropomyosin-deformation dependent crossbridge unbinding rates significantly affects our results for *k*_tr_. Firstly, the Ca^2+^-dependence is stronger for the case with constant unbinding rate, and better represents experimental observations of 4–8 fold changes, which suggests that the choice of a constant crossbridge unbinding rate may be the more physiological one. However, reproducing physiological relaxation rates requires particularly high unbinding rates (≈ 2/ms in mouse). A potential explanation for this is that velocity dependent effects are important even in isometric tension relaxation due to internal sarcomere shortening [76]. Secondly, the behaviour at low Ca^2+^ is different, with the variable unbinding rate showing increasing *k*_tr_. This can be explained by considering the high energy barrier for a single crossbridge binding to a fully blocked thin filament. Experimental results rarely show this region, most likely due to the experimental noise dominating the near-zero force. Regardless, our results and interpretation suggest a Hill fit as applied previously (e.g. [55]) may not be a suitable choice for these results, as the Ca^2+^-*k*_tr_ curves show a clear theoretical minimum.

The current model is designed to investigate the protein-protein interactions responsible for cooperativity. As a computational model can not capture every detail, we have made a range of assumptions to make the model computationally tractable, such as the regular spacing of fixed myosin crossbridges, homogeneity of tropomyosin properties, the choice of two global TnC buffers, and several techniques for reducing the number of states. For a general model of contraction, the most important limitations in the current formulation of the model is the absence of velocity- and length-dependent effects. The current model uses a very simplified sarcomere geometry with regularly spaced crossbridges which are all capable of binding to actin, and does not include the effects of filament overlap which prevents crossbridges from binding to specific regions on the thin filament, even at resting length. Extending the model to represent length-dependence of tension would require an accurate model of filament overlap and other details of sarcomere geometry. This in turn would require changes to the current Monte Carlo sampling strategies and representative state approach, as currently equivalent states would have different crossbridge binding properties depending on the location of unblocked units on the thin filament. However, with respect to the effects of length-dependent activation, the explicit representation of troponin I in our model makes it especially suitable for testing current hypotheses relating to length-dependent modulation of troponin I [77–79]. The addition of velocity dependence would allow us to investigate the interaction between velocity-dependent unbinding and XB-RU effects in ventricular relaxation. Although spatially detailed velocity dependence would likely be too computationally demanding for a practical model, the current dynamic model framework is suitable for extensions using averaged strain-distortion approaches [80, 81].

To limit model complexity, the current model uses simple two-state crossbridge kinetics in which the transitions which affect tropomyosin deformation are considered to be most relevant. Another possible extension of the model is to incorporate more detailed crossbridge dynamics, using a 3-state (weak, strong, unbound) [35] or more complex model which incorporates ATP/ADP/P_i_ kinetics [82]. Such extensions are not expected to affect the steady-state results, as our current model can be interpreted as a steady-state approximation of a more complex modelling framework. These extensions could significantly affect dynamic model results and would also allow for more detailed velocity-dependent binding rates between different crossbridge states. Extensions with ATP kinetics would also allow for more quantitative comparison with experimental data showing ATP-dependence of force, as shown in the rigor crossbridge tests in Fig 6B. However, spatially detailed effects would have to be simplified for an extended dynamic model to keep such an extended model tractable, using techniques such as those applied for blocked and unblocked TnC states (TnC_U_, TnC_B_) in the current model.

Finally, the model currently consists of 750 ODEs, which is a relatively high number compared to many phenomenological contraction models currently available. Models which explicitly represent interactions between RU’s range in complexity from a few ODEs to several thousands. For example, the work by Razumova et al. uses the assumptions of a uniform spatial distribution of RU states, which allows them to reproduce cooperative effects with a lower number of ODEs [83]. However, cooperative activation of RU’s breaks this assumption as unblocked units tend to cluster together. Furthermore, states with identical number of unblocked RU’s can have very different crossbridge binding properties (see Fig 2). More recent work from this group no longer assumes a uniform spatial distribution, but requires several thousand ODEs to represent 9 RU’s [38], as does the model by Dobrunz et al. which takes a similar approach [84]. Although a high number of ODEs has been used before in whole organ models [39], and is thus not an immediate computational problem, it limits their portability and ease of use. Thus, model reduction strategies will form an important part of future work. A potential advantage of our modelling approach is its basis in an underlying biophysical model of the tropomyosin chain. This approach allows for longer-range interactions than these previous ODE models including only nearest-neighbour interaction, and results in fewer free parameters compared to approaches where RU-RU, XB-RU and XB-XB effects are defined by independent parameters. A potential disadvantage of our model is the requirement for a pre-calculation using Monte-Carlo sampling, which is computationally costly. To increase the models usability, an implementation including the results of the Monte Carlo sampling procedures are made available online at cemrg.co.uk. Future work in both model reduction and deformation-dependence will further increase the usability of this biophysically detailed model in a whole-organ setting, and improve the predictive power of multi-scale cardiac models in biological and clinical applications.

## Supporting Information

S1 Text Supporting information with model development details. The supporting information describes: (1) Details for the model for tropomyosin as a flexible chain and the finite element solution of these equations. (2) Details for the derivation of Eq 5 for the tropomyosin state energy. (3) The Monte Carlo sampling algorithm used to approximate the result of Eq 8. (4) Details of force calculation and Hill curve fitting. (5) A description of the experimental data used for rat and human calcium transients.
(PDF)

S1 Fig Effect of spatial arrangement of the thin filament. This figure demonstrates the importance of the spatial arrangement of the thin filament in the continuous flexible chain model. The left column represents the normal 38.5 nm spacing on which all our models are based. The middle column uses half spacing (19.25 nm), effectively halving the width of an RU, but shows more RU’s unblocked to result in an identical length of the tropomyosin chain being unblocked. The right column also uses half spacing and shows an identical number of 5 RU’s unblocked as the left column. All differences in free energy Δ*E* are relative to the fully blocked state shown in the top plot in each column. There is a minor difference in the energy freed in unblocking between the left and middle column (A to D vs B to E) due to effect of the spacing of the blocked RU’s flanking the unblocked region. However, this effect is significantly smaller than the effect of differences in RU size when an identical number of smaller size RU’s are unblocked (e.g. compare D and F). Crossbridge binding is even more similar in the left and middle column (D to G vs E to H) with both changes the free energy by around 1.6*k*_*B*_ *T*, while in the right column (F to I) the difference is 5*k*_*B*_ *T*. Thus, the spatial arrangement of the thin filament in the flexible chain model is more influential than the particular number of RU’s, because flexible chain models of tropomyosin are not limited to nearest-neighbour interactions. Note that the closer spacing does not represent any particular physiological condition, as troponin spacing is highly conserved across species.
(TIF)

S2 Fig Description of representative classes and states. The left plot shows the variation in free energy within each representative class, which is at most 0.11%. The right plot shows the number of states within each class, varying from 1 to 1.2 million. There is a clear tendency for classes with lower energy to have fewer states in each class, as these correspond to longer connected unblocked regions, leading to fewer possible locations within 26 RU. However, due to the symmetries in certain states, classes with a low number of states appear along the entire energy range. Notably, other than the ‘fully blocked’ and ‘fully unblocked’ states, which each have their own class, so do the two additional stats UUUUUUUUBUUUUUUUUBUUUUUUUU, and UUBUUBUUBUUBUUBUUBUUBUUBUU, due to this kind of symmetry.
(TIF)

## References

[1] Sun YB, Lou F, Irving M (2009) Calcium- and myosin-dependent changes in troponin structure during activation of heart muscle. The Journal of Physiology 587: 155–163. 10.1113/jphysiol.2008.164707 19015190 PMC2670030

[2] Gao WD, Perez NG, Marban E (1998) Calcium cycling and contractile activation in intact mouse cardiac muscle. The Journal of Physiology 507: 175–184. 10.1111/j.1469-7793.1998.175bu.x 9490835 PMC2230761

[3] Rice JJ, Winslow RL, Hunter WC (1999) Comparison of putative cooperative mechanisms in cardiac muscle: length dependence and dynamic responses. American Journal of Physiology—Heart and Circulatory Physiology 276: H1734–H1754. 10.1152/ajpheart.1999.276.5.H1734 10330260

[4] Trayanova NA, Rice JJ (2011) Cardiac electromechanical models: From cell to organ. Frontiers in Physiology 2. 10.3389/fphys.2011.00043 PMC3154390 21886622

[5] Campbell KS (2014) Dynamic coupling of regulated binding sites and cycling myosin heads in striated muscle. The Journal of General Physiology 143: 387–399. 10.1085/jgp.201311078 24516189 PMC3933939

[6] Gordon AM, Homsher E, Regnier M (2000) Regulation of contraction in striated muscle. Physiological Reviews 80: 853–924. 10747208 10.1152/physrev.2000.80.2.853

[7] Pan BS, Gordon AM, Luo ZX (1989) Removal of tropomyosin overlap modifies cooperative binding of myosin s-1 to reconstituted thin filaments of rabbit striated muscle. The Journal of biological chemistry 264: 8495–8498. 2722785

[8] Gong H, Hatch V, Ali L, Lehman W, Craig R, et al (2005) Mini-thin filaments regulated by troponin–tropomyosin. Proceedings of the National Academy of Sciences of the United States of America 102: 656–661. 10.1073/pnas.0407225102 15644437 PMC545539

[9] Solaro RJ, Henze M, Kobayashi T (2013) Integration of troponin I phosphorylation with cardiac regulatory networks. Circulation Research 112: 355–366. 10.1161/CIRCRESAHA.112.268672 23329791 PMC3567448

[10] Gillis TE, Martyn DA, Rivera AJ, Regnier M (2007) Investigation of thin filament near-neighbour regulatory unit interactions during force development in skinned cardiac and skeletal muscle. The Journal of Physiology 580: 561–576. 10.1113/jphysiol.2007.128975 17317743 PMC2075566

[11] Rao VS, Marongelli EN, Guilford WH (2009) Phosphorylation of tropomyosin extends cooperative binding of myosin beyond a single regulatory unit. Cell Motility and the Cytoskeleton 66: 10–23. 10.1002/cm.20321 18985725 PMC2770177

[12] Trybus KM, Taylor EW (1980) Kinetic studies of the cooperative binding of subfragment 1 to regulated actin. Proceedings of the National Academy of Sciences of the United States of America 77: 7209–7213. 10.1073/pnas.77.12.7209 6938966 PMC350471

[13] Campbell KB, Razumova MV, Kirkpatrick RD, Slinker BK (2001) Nonlinear myofilament regulatory processes affect frequency-dependent muscle fiber stiffness. Biophysical Journal 81: 2278–2296. 10.1016/S0006-3495(01)75875-4 11566798 PMC1301699

[14] Tanner B, Daniel T, Regnier M (2012) Filament compliance influences cooperative activation of thin filaments and the dynamics of force production in skeletal muscle. PLoS Computational Biology 8. 10.1371/journal.pcbi.1002506 PMC3349719 22589710

[15] Smith L, Tainter C, Regnier M, Martyn D (2009) Cooperative cross-bridge activation of thin filaments contributes to the frank-starling mechanism in cardiac muscle. Biophysical Journal 96: 3692–3702. 10.1016/j.bpj.2009.02.018 19413974 PMC3325146

[16] Dou Y, Arlock P, Arner A (2007) Blebbistatin specifically inhibits actin-myosin interaction in mouse cardiac muscle. American Journal of Physiology Cell Physiology 293: C1148–1153. 10.1152/ajpcell.00551.2006 17615158

[17] Farman G, Allen E, Schoenfelt K, Backx P, De Tombe P (2010) The role of thin filament cooperativity in cardiac length-dependent calcium activation. Biophysical Journal 99: 2978–2986. 10.1016/j.bpj.2010.09.003 21044595 PMC2965940

[18] Butters CA, Tobacman JB, Tobacman LS (1997) Cooperative effect of calcium binding to adjacent troponin molecules on the thin filament-myosin subfragment 1 MgATPase rate. Journal of Biological Chemistry 272: 13196–13202. 10.1074/jbc.272.20.13196 9148936

[19] Davis JP, Norman C, Kobayashi T, Solaro RJ, Swartz DR, et al (2007) Effects of thin and thick filament proteins on calcium binding and exchange with cardiac troponin c. Biophysical Journal 92: 3195–3206. 10.1529/biophysj.106.095406 17293397 PMC1852344

[20] Hannon JD, Martyn DA, Gordon AM (1992) Effects of cycling and rigor crossbridges on the conformation of cardiac troponin c. Circulation Research 71: 984–991. 10.1161/01.RES.71.4.984 1516169

[21] Kentish JC, Wrzosek A (1998) Changes in force and cytosolic Ca2+ concentration after length changes in isolated rat ventricular trabeculae. The Journal of Physiology 506: 431–444. 10.1111/j.1469-7793.1998.431bw.x 9490870 PMC2230716

[22] Bers D (2001) Excitation-Contraction Coupling and Cardiac Contractile Force. Springer, 2nd ed. edition.

[23] Boussouf SE, Geeves MA (2007) Tropomyosin and troponin cooperativity on the thin filament In: Ebashi S, Ohtsuki I, editors, Regulatory Mechanisms of Striated Muscle Contraction, Springer Japan, number 592 in Advances in Experimental Medicine and Biology. pp. 99–109. 10.1007/978-4-431-38453-3_10 17278359

[24] Galińska-Rakoczy A, Engel P, Xu C, Jung H, Craig R, et al (2008) Structural basis for the regulation of muscle contraction by troponin and tropomyosin. Journal of Molecular Biology 379: 929–935. 10.1016/j.jmb.2008.04.062 18514658 PMC2483953

[25] Vinogradova MV, Stone DB, Malanina GG, Karatzaferi C, Cooke R, et al (2005) Ca2+-regulated structural changes in troponin. Proceedings of the National Academy of Sciences of the United States of America 102: 5038–5043. 10.1073/pnas.0408882102 15784741 PMC555973

[26] Pirani A, Vinogradova MV, Curmi PMG, King WA, Fletterick RJ, et al (2006) An atomic model of the thin filament in the relaxed and ca2+-activated states. Journal of Molecular Biology 357: 707–717. 10.1016/j.jmb.2005.12.050 16469331

[27] Rieck DC, Li KL, Ouyang Y, Solaro RJ, Dong WJ (2013) Structural basis for the in situ Ca(2+) sensitization of cardiac troponin c by positive feedback from force-generating myosin cross-bridges. Archives of biochemistry and biophysics 537: 198–209. 10.1016/j.abb.2013.07.013 23896515 PMC3836555

[28] Solaro RJ, Rosevear P, Kobayashi T (2008) The unique functions of cardiac troponin I in the control of cardiac muscle contraction and relaxation. Biochemical and biophysical research communications 369: 82–87. 10.1016/j.bbrc.2007.12.114 18162178 PMC2365727

[29] Konhilas JP, Irving TC, Wolska BM, Jweied EE, Martin AF, et al (2003) Troponin I in the murine myocardium: influence on length-dependent activation and interfilament spacing. The Journal of Physiology 547: 951–961. 10.1113/jphysiol.2002.038117 12562915 PMC2342721

[30] Layland J, Solaro RJ, Shah AM (2005) Regulation of cardiac contractile function by troponin i phosphorylation. Cardiovascular Research 66: 12–21. 10.1016/j.cardiores.2004.12.022 15769444

[31] McKillop DF, Geeves MA (1993) Regulation of the interaction between actin and myosin subfragment 1: evidence for three states of the thin filament. Biophysical journal 65: 693–701. 10.1016/S0006-3495(93)81110-X 8218897 PMC1225772

[32] Rice JJ, Tu Y, Poggesi C, De Tombe PP (2008) Spatially-compressed cardiac myofilament models generate hysteresis that is not found in real muscle. Pacific Symposium on Biocomputing: 366–377. 18229700

[33] Schneider NS, Shimayoshi T, Amano A, Matsuda T (2006) Mechanism of the frank-starling law–a simulation study with a novel cardiac muscle contraction model that includes titin and troponin i. Journal of Molecular and Cellular Cardiology 41: 522–536. 10.1016/j.yjmcc.2006.06.003 16860336

[34] Niederer SA, Hunter PJ, Smith NP (2006) A quantitative analysis of cardiac myocyte relaxation: a simulation study. Biophysical journal 90: 1697–1722. 10.1529/biophysj.105.069534 16339881 PMC1367320

[35] Rice JJ, Wang F, Bers DM, De Tombe PP (2008) Approximate model of cooperative activation and crossbridge cycling in cardiac muscle using ordinary differential equations. Biophysical Journal 95: 2368–2390. 10.1529/biophysj.107.119487 18234826 PMC2517033

[36] Land S, Niederer SA, Aronsen JM, Espe EKS, Zhang L, et al (2012) An analysis of deformation-dependent electromechanical coupling in the mouse heart. The Journal of Physiology 590: 4553–4569. 10.1113/jphysiol.2012.231928 22615436 PMC3477757

[37] Rice JJ, Stolovitzky G, Tu Y, de Tombe PP (2003) Ising model of cardiac thin filament activation with nearest-neighbor cooperative interactions. Biophysical Journal 84: 897–909. 10.1016/S0006-3495(03)74907-8 12547772 PMC1302668

[38] Campbell SG, Lionetti FV, Campbell KS, McCulloch AD (2010) Coupling of adjacent tropomyosins enhances cross-bridge-mediated cooperative activation in a markov model of the cardiac thin filament. Biophysical Journal 98: 2254–2264. 10.1016/j.bpj.2010.02.010 20483334 PMC2872217

[39] Sheikh F, Ouyang K, Campbell SG, Lyon RC, Chuang J, et al (2012) Mouse and computational models link mlc2v dephosphorylation to altered myosin kinetics in early cardiac disease. Journal of Clinical Investigation 122: 1209–1221. 10.1172/JCI61134 22426213 PMC3314469

[40] Smith D, Maytum R, Geeves M (2003) Cooperative regulation of myosin-actin interactions by a continuous flexible chain I: Actin-tropomyosin systems. Biophysical Journal 84: 3155–3167. 10.1016/S0006-3495(03)70040-X 12719245 PMC1302876

[41] Smith D, Geeves M (2003) Cooperative regulation of myosin-actin interactions by a continuous flexible chain II: actin-tropomyosin-troponin and regulation by calcium. Biophysical Journal 84: 3168–3180. 10.1016/S0006-3495(03)70041-1 12719246 PMC1302877

[42] Mijailovich SM, Kayser-Herold O, Li X, Griffiths H, Geeves MA (2012) Cooperative regulation of myosin-s1 binding to actin filaments by a continuous flexible Tm–Tn chain. European Biophysics Journal 41: 1015–1032. 10.1007/s00249-012-0859-8 23052974 PMC3509328

[43] Metalnikova NA, Tsaturyan AK (2013) A mechanistic model of ca regulation of thin filaments in cardiac muscle. Biophysical Journal 105: 941–950. 10.1016/j.bpj.2013.06.044 23972846 PMC3752105

[44] Howard J (2001) Mechanics of motor proteins and the cytoskeleton. Sinauer Associates Inc, US.

[45] Metzger JM (1995) Myosin binding-induced cooperative activation of the thin filament in cardiac myocytes and skeletal muscle fibers. Biophysical Journal 68: 1430–1442. 10.1016/S0006-3495(95)80316-4 7787029 PMC1282038

[46] Brandt PW, Diamond MS, Schachat FH (1984) The thin filament of vertebrate skeletal muscle co-operatively activates as a unit. Journal of Molecular Biology 180: 379–384. 10.1016/S0022-2836(84)80010-8 6542594

[47] Vibert P, Craig R, Lehman W (1997) Steric-model for activation of muscle thin filaments. Journal of molecular biology 266: 8–14. 10.1006/jmbi.1996.0800 9054965

[48] Poole KJV, Lorenz M, Evans G, Rosenbaum G, Pirani A, et al (2006) A comparison of muscle thin filament models obtained from electron microscopy reconstructions and low-angle x-ray fibre diagrams from non-overlap muscle. Journal of Structural Biology 155: 273–284. 10.1016/j.jsb.2006.02.020 16793285

[49] Bershitsky SY, Ferenczi MA, Koubassova NA, Tsaturyan AK (2009) Insight into the actin-myosin motor from x-ray diffraction on muscle. Frontiers in Bioscience (Landmark Edition) 14: 3188–3213. 10.2741/3444 19273266

[50] Sommese RF, Sung J, Nag S, Sutton S, Deacon JC, et al (2013) Molecular consequences of the R453C hypertrophic cardiomyopathy mutation on human *β*-cardiac myosin motor function. Proceedings of the National Academy of Sciences 110: 12607–12612. 10.1073/pnas.1309493110 PMC3732973 23798412

[51] He ZH, Bottinelli R, Pellegrino MA, Ferenczi MA, Reggiani C (2000) ATP consumption and efficiency of human single muscle fibers with different myosin isoform composition. Biophysical Journal 79: 945–961. 10.1016/S0006-3495(00)76349-1 10920025 PMC1300991

[52] McDonald KS, Moss RL (1995) Osmotic compression of single cardiac myocytes eliminates the reduction in Ca2+ sensitivity of tension at short sarcomere length. Circ Res 77: 199–205. 10.1161/01.RES.77.1.199 7788878

[53] Dobesh DP, Konhilas JP, de Tombe PP (2002) Cooperative activation in cardiac muscle: impact of sarcomere length. American Journal of Physiology—Heart and Circulatory Physiology 282: H1055–H1062. 10.1152/ajpheart.00667.2001 11834504

[54] Sun YB, Irving M (2010) The molecular basis of the steep force-calcium relation in heart muscle. Journal of Molecular and Cellular Cardiology 48: 859–865. 10.1016/j.yjmcc.2009.11.019 20004664 PMC2860225

[55] Regnier M, Martin H, Barsotti R, Rivera A, Martyn D, et al (2004) Cross-bridge versus thin filament contributions to the level and rate of force development in cardiac muscle. Biophysical Journal 87: 1815–1824. 10.1529/biophysj.103.039123 15345560 PMC1304586

[56] Little SC, Biesiadecki BJ, Kilic A, Higgins RSD, Janssen PML, et al (2012) The rates of ca2+ dissociation and cross-bridge detachment from ventricular myofibrils as reported by a fluorescent cardiac troponin c. Journal of Biological Chemistry 287: 27930–27940. 10.1074/jbc.M111.337295 22718768 PMC3431663

[57] Stull LB, Leppo MK, MarbÃ¡n E, Janssen PML (2002) Physiological determinants of contractile force generation and calcium handling in mouse myocardium. Journal of Molecular and Cellular Cardiology 34: 1367–1376. 10.1006/jmcc.2002.2065 12392997

[58] Stull LB, Hiranandani N, Kelley MA, Leppo MK, MarbÃ¡n E, et al (2006) Murine strain differences in contractile function are temperature- and frequency-dependent. PflÃ¼gers Archiv—European Journal of Physiology 452: 140–145. 10.1007/s00424-005-0020-y 16397793

[59] Bluhm WF, Meyer M, Sayen M, Swanson EA, Dillmann WH (1999) Overexpression of sarcoplasmic reticulum Ca2+–ATPase improves cardiac contractile function in hypothyroid mice. Cardiovascular Research 43: 382–388. 10.1016/S0008-6363(99)00109-1 10536668

[60] Bluhm WF, Kranias EG, Dillmann WH, Meyer M (2000) Phospholamban: a major determinant of the cardiac force-frequency relationship. American Journal of Physiology—Heart and Circulatory Physiology 278: H249–H255. 10644605 10.1152/ajpheart.2000.278.1.H249

[61] Stuyvers BD, McCulloch AD, Guo J, Duff HJ, ter Keurs HEDJ (2002) Effect of stimulation rate, sarcomere length and ca2+ on force generation by mouse cardiac muscle. The Journal of Physiology 544: 817–830. 10.1113/jphysiol.2002.024430 12411526 PMC2290620

[62] Land S, Niederer SA, Louch WE, Røe AT, Aronsen JM, et al (2014) Computational modeling of Takotsubo cardiomyopathy: effect of spatially varying *β*-adrenergic stimulation in the rat left ventricle. American Journal of Physiology—Heart and Circulatory Physiology 307: H1487–H1496. 10.1152/ajpheart.00443.2014 25239804 PMC4233305

[63] Mulieri LA, Hasenfuss G, Leavitt B, Allen PD, Alpert NR (1992) Altered myocardial force-frequency relation in human heart failure. Circulation 85: 1743 10.1161/01.CIR.85.5.1743 1572031

[64] Pieske B, SÃ¼tterlin M, Schmidt-Schweda S, Minami K, Meyer M, et al (1996) Diminished post-rest potentiation of contractile force in human dilated cardiomyopathy. functional evidence for alterations in intracellular Ca2+ handling. Journal of Clinical Investigation 98: 764–776. 8698869 10.1172/JCI118849 PMC507487

[65] Land S, Louch WE, Niederer SA, Aronsen JM, Christensen GA, et al (2013) Beta-adrenergic stimulation maintains cardiac function in Serca2 knockout mice. Biophysical Journal 104: 1349–1356. 10.1016/j.bpj.2013.01.042 23528094 PMC3602781

[66] Louch WE, Sheehan KA, Wolska BM (2011) Methods in cardiomyocyte isolation, culture, and gene transfer. Journal of Molecular and Cellular Cardiology 51: 288–298. 10.1016/j.yjmcc.2011.06.012 21723873 PMC3164875

[67] TÃ¸ndel K, Land S, Niederer SA, Smith NP (2015) Quantifying inter-species differences in contractile function through biophysical modelling. The Journal of Physiology 593: 1083–1111. 10.1113/jphysiol.2014.279232 25480801 PMC4358673

[68] Coppini R, Ferrantini C, Yao L, Fan P, Del Lungo M, et al (2013) Late sodium current inhibition reverses electromechanical dysfunction in human hypertrophic cardiomyopathy. Circulation 127: 575–584. 10.1161/CIRCULATIONAHA.112.134932 23271797

[69] Hunter PJ, McCulloch AD, Ter Keurs H (1998) Modelling the mechanical properties of cardiac muscle. Progress in biophysics and molecular biology 69: 289–331. 10.1016/S0079-6107(98)00013-3 9785944

[70] Land S, Niederer SA, Smith NP (2012) Efficient computational methods for strongly coupled cardiac electromechanics. Biomedical Engineering, IEEE Transactions on 59: 1219–1228. 10.1109/TBME.2011.2112359 21303740

[71] Patel JR, Fitzsimons DP, Buck SH, Muthuchamy M, Wieczorek DF, et al (2001) PKA accelerates rate of force development in murine skinned myocardium expressing *α*- or *β*-tropomyosin. American Journal of Physiology—Heart and Circulatory Physiology 280: H2732–H2739. 11356630 10.1152/ajpheart.2001.280.6.H2732

[72] Fitzsimons DP, Patel JR, Campbell KS, Moss RL (2001) Cooperative mechanisms in the activation dependence of the rate of force development in rabbit skinned skeletal muscle fibers. The Journal of General Physiology 117: 133–148. 10.1085/jgp.117.2.133 11158166 PMC2217243

[73] Palmer S, Kentish JC (1998) Roles of Ca2+ and crossbridge kinetics in determining the maximum rates of Ca2+ activation and relaxation in rat and guinea pig skinned trabeculae. Circulation Research 83: 179–186. 10.1161/01.RES.83.2.179 9686757

[74] Wolff MR, McDonald KS, Moss RL (1995) Rate of tension development in cardiac muscle varies with level of activator calcium. Circulation Research 76: 154–160. 10.1161/01.RES.76.1.154 8001274

[75] Moss RL, Razumova M, Fitzsimons DP (2004) Myosin crossbridge activation of cardiac thin filaments implications for myocardial function in health and disease. Circulation Research 94: 1290–1300. 10.1161/01.RES.0000127125.61647.4F 15166116

[76] ter Keurs HE, Rijnsburger WH, van Heuningen R, Nagelsmit MJ (1980) Tension development and sarcomere length in rat cardiac trabeculae. evidence of length-dependent activation. Circulation Research 46: 703–714. 7363419 10.1161/01.res.46.5.703

[77] Terui T, Sodnomtseren M, Matsuba D, Udaka J, Ishiwata S, et al (2008) Troponin and titin coordinately regulate length-dependent activation in skinned porcine ventricular muscle. The Journal of General Physiology 131: 275–283. 10.1085/jgp.200709895 18299397 PMC2248715

[78] Fukuda N, Terui T, Ohtsuki I, Ishiwata S, Kurihara S (2009) Titin and troponin: Central players in the frank-starling mechanism of the heart. Current Cardiology Reviews 5: 119–124. 10.2174/157340309788166714 20436852 PMC2805814

[79] Wijnker PJM, Sequeira V, Foster DB, Li Y, Remedios CGd, et al (2014) Length-dependent activation is modulated by cardiac troponin I bisphosphorylation at Ser23 and Ser24 but not by Thr143 phosphorylation. American Journal of Physiology—Heart and Circulatory Physiology 306: H1171–H1181. 10.1152/ajpheart.00580.2013 24585778 PMC3989756

[80] Razumova MV, Bukatina AE, Campbell KB (1999) Stiffness-distortion sarcomere model for muscle simulation. J Appl Physiol 87: 1861–1876. 10562631 10.1152/jappl.1999.87.5.1861

[81] Ford SJ, Chandra M, Mamidi R, Dong W, Campbell KB (2010) Model representation of the nonlinear step response in cardiac muscle. The Journal of General Physiology 136: 159–177. 10.1085/jgp.201010467 20660660 PMC2912065

[82] Bickham DC, West TG, Webb MR, Woledge RC, Curtin NA, et al (2011) Millisecond-scale biochemical response to change in strain. Biophysical Journal 101: 2445–2454. 10.1016/j.bpj.2011.10.007 22098743 PMC3218346

[83] Razumova MV, Bukatina AE, Campbell KB (2000) Different Myofilament Nearest-Neighbor Interactions Have Distinctive Effects on Contractile Behavior. Biophysical Journal 78: 3120–3137. 10.1016/S0006-3495(00)76849-4 10827989 PMC1300894

[84] Dobrunz LE, Backx PH, Yue DT (1995) Steady-state [Ca2+]i-force relationship in intact twitching cardiac muscle: direct evidence for modulation by isoproterenol and EMD 53998. Biophysical Journal 69: 189–201. 10.1016/S0006-3495(95)79889-7 7669896 PMC1236237

