# Supplementary material for: A Spatially Detailed Model of Isometric Contraction Based on Competitive Binding of Troponin I Explains Cooperative Interactions between Tropomyosin and Crossbridges
Source: PLoS Comput Biol. 2015 Aug 11;11(8):e1004376. doi: 10.1371/journal.pcbi.1004376 (PMC4532474; doi:10.1371/journal.pcbi.1004376)
Supplement: S1 Text — The supporting information describes: (1) Details for the model for tropomyosin as a flexible chain and the finite element solution of these equations. (2) Details for the derivation of Eq 5 for the tropomyosin state energy. (3) The Monte Carlo sampling algorithm used to approximate the result of Eq 8. (4) Details of force calculation and Hill curve fitting. (5) A description of the experimental data used for rat and human calcium transients. (PDF) [file pcbi.1004376.s001.pdf]

# Supporting Information S1 Text

## A spatially detailed model of isometric contraction based on competitive binding of troponin I explains cooperative interactions between tropomyosin and crossbridges

Sander Land Steven A. Niederer

## Model for tropomyosin as a flexible chain

The models developed in this paper are based on a flexible chain model of tropomyosin, originally developed by Smith and Geeves [40]. Specifically, we use the modification proposed by Metalnikova and Tsaturyan, which takes into account the helical twist of the tropomyosin filament [43]. In this model, the free energy of a tropomyosin chain is given by:

$$E_{\mathrm{tm}}(\phi(x)) = \frac{1}{2}\int_0^l \alpha\phi(x)^2 + K\left(\left(\frac{a\phi''(x)}{\sqrt{g_0}}\right)^2 + \left(2a\psi\phi'(x)\right)^2\right)\,\mathrm{d}x \tag{1}$$

Where $\phi$ is the angle of displacement in radians, $a = 4.2\,\mathrm{nm}$ is the radius the tropomyosin helix, $\psi = 2\pi/72\,\mathrm{nm} = 0.0873/\mathrm{nm}$ is the helical twist of a tropomyosin chain, $\alpha$ is strength of the electrostatic actin-Tm interaction, $K$ is the bending stiffness of tropomyosin. Note that as the derivation of a non-dimensional variant of the model in previous work has an error (Equation 4 to Equation 5 in [43]), we use the original formulation. As a starting point for parametrization, we initially fitted the two free parameters to their three reported non-dimensional values $\beta = 2a\psi^2\sqrt{Kg_0/\alpha} \approx 3.5$ $\xi = 4Ka^2/\left(\alpha\sqrt{g_0}\right)^{1/4} \approx 19.25$, $G = \alpha\xi(25/180\pi)^2\sqrt{g_0(1+\beta)}/k_BT \approx 4$, resulting in $\alpha = 2.15$ pN, $K = 4550$ pN nm$^2$.
These parameters are not directly suitable, as they result in very low cooperativity more typical of skinned muscle cells. Due to computational constraints, the ratio between the two free parameters $\alpha$ and $K$ is kept constant throughout our investigation. The magnitude of the parameters $\alpha$ and $K$ are varied by the parameter $\gamma$, defined by $\alpha = 2.15\gamma$ pN, $K = 4550\gamma$ pN nm$^2$. This scaling allows us to investigate the effect of the energy parameters on cooperativity, and reproduce the steep force-calcium relationships seen in intact muscle. Keeping the ratio constant results in $E_{\mathrm{tm}}(\phi) \sim \gamma$, allowing the magnitude of these parameters to be investigated without recalculating the solution for equation 1.

The displacement $\phi(x)$ for given boundary conditions $\phi(x_i) = \phi_i$ is given by the minimal energy solution to equation 1, which obeys:

$$E(\phi(x) + \delta\phi(x)) - E(\phi(x)) = 0 \tag{2}$$

Where $\delta\phi(x)$ is an infinitesimal variation in the angle of displacement. Using $\delta\phi(x)^2 = 0$ results in:

$$\int_0^l \alpha\phi(x)\delta\phi(x) + \left(Ka^2/g_0\right)\phi''(x)\delta\phi''(x) + \left(4a^2\psi^2K\right)\phi'(x)\delta\phi'(x)\,\mathrm{d}x = 0 \tag{3}$$

Introducing basis functions $\beta_1(x), \beta_2(x), \ldots$, writing the angle as $\phi = \sum b_i\beta_i(x)$ and using the test function $\delta\phi = \beta_j(x)$ results in:

$$\forall\beta_j \;:\; \int_0^l \alpha\sum_i b_i\beta_i(x)\beta_j(x) + \left(Ka^2/g_0\right)\sum_i b_i\beta_i''(x)\beta_j''(x) + \left(4Ka^2\psi^2\right)\sum_i b_i\beta_i'(x)\beta_j'(x)\,\mathrm{d}x = 0 \tag{4}$$

Which reduces to the matrix-vector problem:

$$\mathbf{A}\mathbf{b} = 0 \tag{5}$$

$$A_{ij} = \alpha \int_0^l \beta_i(x)\beta_j(x)\; dx + \Big(Ka^2/g_0\Big) \int_0^l \beta_i''(x)\beta_j''(x)\; \mathrm{d}x + \Big(4Ka^2\psi^2\Big) \int_0^l \beta_i'(x)\beta_j'(x)\; \mathrm{d}x \tag{6}$$

$$\mathbf{b} = (b_1, b_2, \dots) \tag{7}$$

Where the integrals are calculated using Gaussian integration with 5 points per element. The matrix $\mathbf{A}$ is only determined once, and different configurations of the thin filament are solved by applying different Dirichlet boundary conditions to a copy of the matrix $\mathbf{A}$.

Our finite element model used one-dimensional cubic Hermite basis functions to ensure both a continuous solution on the entire domain and the presence of a well-defined second derivative $\beta''$. We use a mesh resolution of 0.5 nm which is sufficient for a converged solution and allows boundary conditions to be prescribed on nodes. The potential sites for pinning of tropomyosin to actin by troponin I are set to be evenly spaced 38.5 nm apart consistent with structural data on actin and troponin [46]. Any active pinning sites $x_i$ are implemented by the finite element Dirichlet boundary condition $\phi(x_i) = -25\pi/180$ (25° converted to radians). The influence of the boundary conditions at the filament ends was ensured to be minimal by implementing the filament ends as five additional pinned sites spaced at the normal 38.5 nm distance, which is sufficient to give identical free energy for the solution with any single unblocked site, thus minimizing the effect of boundary conditions on unblocking kinetics. For the model including crossbridges we introduce 69 evenly spaced binding sites 14.5 nm apart based on x-ray diffraction data [49], with potential Dirichlet boundary conditions $\phi(x_i) = +10\pi/180$. If cross-bridge binding site is identical to troponin pinning site, the crossbridge binding site is shifted to the next node (0.5 nm) to avoid numerical problems, although in practice the free energy $E_{\mathrm{tm}}$ of these solutions is very high, and numerically results in $e^{-\frac{E_{\mathrm{tm}}}{k_B T}} \approx 0$. S1 Fig. shows some example solutions of the continuous flexible chain model with energy differences, illustrating the importance of spatial arrangement and pinning locations.

## Derivation for the sum of Boltzmann terms for a tropomyosin state

This section extends the calculation of the tropomyosin state energy (Equation 5 given in section on "Steady-state model of thin filament kinetics"). The total free energy of a thin filament was previously mentioned as:

$$E_{\text{tot}}(i,j,k,l) = E_{\text{tm}} + (n-i)E_A + jE_M + k(E_I + E_C) + lE_C \tag{8}$$

Where $n$ is the number of RU's, $i = N_u(\text{tm})$ the number of unblocked RU's (and thus $(n-i)$ the number of blocked RU's), $j = N_{\text{xb}}(\text{tm})$ the number of crossbridges bound, $k$ is the number of RU's in the TnI·TnC·$Ca^{2+}$ state, and $l$ is the number of RU's in the TnC·$Ca^{2+}$ state without TnI bound.

Using the binomial theorem twice along with some algebraic manipulation, the sum of Boltzmann terms for the thin filament being in state tm is derived as:

$$\begin{aligned}
P(\text{tm}) &\sim \sum_{k=0}^{i}\sum_{l=0}^{n-k}\binom{i}{k}\binom{n-k}{l}e^{-\frac{E_{\text{tot}}(i,j,k,l)}{k_BT}} \qquad \text{where} \quad i = N_u(\text{tm}), j = N_{\text{xb}}(\text{tm}) \\
&= \sum_{k=0}^{i}\sum_{l=0}^{n-k}\binom{i}{k}\binom{n-k}{l}e^{-\frac{E_{\text{tm}}+(n-i)E_A+jE_m+k(E_I+E_C)+lE_C}{k_BT}} \\
&= e^{-\frac{E_{\text{tm}}+(n-i)E_A+jE_M}{k_BT}}\sum_{k=0}^{i}\binom{i}{k}e^{-\frac{k(E_I+E_C)}{k_BT}}\sum_{l=0}^{n-k}\binom{n-k}{l}e^{-\frac{lE_C}{k_BT}} \\
&= e^{-\frac{E_{\text{tm}}+(n-i)E_A+jE_M}{k_BT}}\sum_{k=0}^{i}\binom{i}{k}e^{-\frac{k(E_I+E_C)}{k_BT}}\overbrace{\sum_{l=0}^{n-k}\binom{n-k}{l}\left(e^{-\frac{E_C}{k_BT}}\right)^{l}1^{(n-k)-l}}^{\text{binomial theorem form}} \\
&= e^{-\frac{E_{\text{tm}}+(n-i)E_A+jE_M}{k_BT}}\sum_{k=0}^{i}\binom{i}{k}e^{-\frac{k(E_I+E_C)}{k_BT}}\left(1+e^{-\frac{E_C}{k_BT}}\right)^{n-k} \\
&= e^{-\frac{E_{\text{tm}}+(n-i)E_A+jE_M}{k_BT}}\sum_{k=0}^{i}\binom{i}{k}\left(e^{-\frac{(E_I+E_C)}{k_BT}}\right)^{k}\left(1+e^{-\frac{E_C}{k_BT}}\right)^{n-i}\left(1+e^{-\frac{E_C}{k_BT}}\right)^{i-k} \\
&= e^{-\frac{E_{\text{tm}}+(n-i)E_A+jE_M}{k_BT}}\left(1+e^{-\frac{E_C}{k_BT}}\right)^{n-i}\overbrace{\sum_{k=0}^{i}\binom{i}{k}\left(e^{-\frac{(E_I+E_C)}{k_BT}}\right)^{k}\left(1+e^{-\frac{E_C}{k_BT}}\right)^{i-k}}^{\text{binomial theorem form}} \\
&= e^{-\frac{E_{\text{tm}}+(n-i)E_A+jE_M}{k_BT}}\left(1+e^{-\frac{E_C}{k_BT}}\right)^{n-i}\left(1+e^{-\frac{E_C}{k_BT}}+e^{-\frac{E_I+E_C}{k_BT}}\right)^{i} \\
&= e^{-\frac{E_{\text{tm}}}{k_BT}}\left(e^{-\frac{E_A}{k_BT}}\right)^{(n-i)}\left(e^{-\frac{E_M}{k_BT}}\right)^{j}\left(1+e^{-\frac{E_C}{k_BT}}\right)^{n-i}\left(1+e^{-\frac{E_C}{k_BT}}+e^{-\frac{E_I+E_C}{k_BT}}\right)^{i}
\end{aligned} \tag{9}$$

Which, using the relation between the dissociation constants $K_{\text{DA}}, K_{\text{DI}}, K_{\text{DC}}, K_{\text{DM}}$ and differences in free energy $E_A, E_I, E_C, E_M$, is equivalent to:

$$P(\text{tm}) \sim e^{-\frac{E_{\text{tm}}}{k_BT}}\frac{1}{K_{\text{DA}}^{n-i}}\frac{1}{K_{\text{DM}}^{j}}\left(1+\frac{[\text{Ca}^{2+}]}{K_{\text{DC}}}\right)^{n-i}\left(1+\frac{1}{K_{\text{DI}}}\frac{[\text{Ca}^{2+}]}{K_{\text{DC}}}+\frac{[\text{Ca}^{2+}]}{K_{\text{DC}}}\right)^{i} \tag{10}$$

# Monte Carlo sampling algorithm for crossbridge states

This section describes the Monte Carlo sampling algorithm for approximating the sums $\mathrm{SE}_{i,j}$ from Equation 8. Our sampling strategy first limits the set of states to be sampled to exclude the large number of states which are very improbable, i.e. those in which crossbridges bind to blocked RU's. To select the crossbridges to be sampled, we use the independent crossbridge model to select crossbridges near unblocked RU's which can plausibly bind to actin, based on the energy difference $\Delta E(\mathrm{tm}, \mathrm{xb})$. This gives the set $\mathrm{xb}_p$ of crossbridges that can potentially bind to a tropomyosin state tm as:

$$\mathrm{xb}_p(\mathrm{tm}) = \left\{ \mathrm{xb} \mid e^{-\frac{\Delta E(\mathrm{tm},\mathrm{xb})}{k_B T}} > \varepsilon_p \right\} \tag{11}$$

Where $\varepsilon_p = 10^{-6}$ is the threshold where a crossbridge is considered to be potentially able to bind to actin, $\Delta E(\mathrm{tm}, \mathrm{xb})$ is the difference in $E_{\mathrm{tm}}$ with and without a specific crossbridge, and tm is always a tropomyosin state without crossbridges.

Next, we define the set $T_{\mathrm{xb}}(\mathrm{tm}_0, j)$ as all $\binom{\#\mathrm{xb}_p}{j}$ ways[1] to add $j$ crossbridges from the set $\mathrm{xb}_p$ to the tropomyosin state $\mathrm{tm}_0$:

$$T_{\mathrm{xb}}(\mathrm{tm}_0, j) = \{\mathrm{tm} \in \mathrm{TM} \mid \tau_0(\mathrm{tm}) = \mathrm{tm}_0,\ \mathrm{xb}(\mathrm{tm}) \subset \mathrm{xb}_p(\mathrm{tm}_0),\ N_{\mathrm{xb}}(\mathrm{tm}) = j\} \tag{12}$$

Where

- $\tau_0(\mathrm{tm})$ gives the tropomyosin state $\mathrm{tm}_0$ with identical RU state as tm, but no crossbridges bound.
- $\mathrm{xb}(\mathrm{tm})$ gives the set of bound crossbridges for a state t, and $N_{\mathrm{xb}}(\mathrm{tm})$ is the number of bound crossbridges for a tropomyosin state tm.
- TM is the set of all $2^{26+69}$ tropomyosin states.

This set is typically still too large to be able to calculate the energy difference for tropomyosin deformation for all states. Thus, we perform Monte Carlo sampling of this sum by defining a set $R(\mathrm{tm}_{r0}, j)$ as a random sample of size $n_s = 1000$, and approximate the sum of energies by multiplying by the difference in the size of the sampling set $R$ and the full set $T_{\mathrm{xb}}$. However, if the calculation is tractable, as given by the threshold $\#T_{\mathrm{xb}} \leq 5n_s$, we perform the full calculation. More formally:

$$\mathrm{ST}(\mathrm{tm}_{r0}, j) \begin{cases} \approx \dfrac{\binom{\#\mathrm{xb}_p}{j}}{n_s} \displaystyle\sum_{\mathrm{tm} \in R(\mathrm{tm}_{r0}, j)} e^{-\frac{E_{\mathrm{tm}}}{k_B T}} & \text{if } \binom{\#\mathrm{xb}_p}{j} > 5n_s \\ = \displaystyle\sum_{\mathrm{tm} \in T_{\mathrm{xb}}(\mathrm{tm}_{r0}, j)} e^{-\frac{E_{\mathrm{tm}}}{k_B T}} & \text{otherwise} \end{cases} \tag{13}$$

$$\text{where } R(\mathrm{tm}_{r0}, j) = \text{A random subset of size } n_s \text{ from } T_{\mathrm{xb}}(\mathrm{tm}_{r0}, j) \tag{14}$$

$$\tag{15}$$

This sampling assumes that the probability of being in any state with at least one crossbridge bound that is not in the set $\mathrm{xb}_p$ is approximately zero. Furthermore it requires $n_s$ to be large enough such that the average of $e^{-\frac{E_{\mathrm{tm}}}{k_B T}}$ for the set $R$ is approximately the true average over all $\binom{\#\mathrm{xb}_p}{j}$ 'plausible' states. This sampling was performed for all 3010 representative states as defined in the main text (section "Sampling crossbridge states"). As the calcium-dependence is only dependent on the number of unblocked RU's, the terms $\mathrm{SE}_{i,j}$ are determined using:

$$\mathrm{SE}_{i,j} = \sum_{\{\mathrm{tm}_{r0} \in \mathrm{TM}_{r0} \mid N_u(\mathrm{tm}_{r0}) = i\}} \overbrace{\#\{t \mid \tau_r(\mathrm{tm}) = \mathrm{tm}_{r0}\}}^{\text{number of states in a class}} \cdot \mathrm{ST}(\mathrm{tm}_{r0}, j) \tag{16}$$

[1] $\#S$ denotes the number of elements in a set $S$.

Where

- $\tau_r(\text{tm})$ denotes the representative tropomyosin state of the class of states to which the state tm belongs. Classes are defined by connected stretches of unblocked RU's, with their representative states selected as (one of the) states which has a Boltzmann term $e^{-\frac{E_{\text{tm}}}{k_B T}}$ closest to the class mean.
- $\text{TM}_{\text{r0}} = \tau_r(\tau_0(\text{TM}))$ is the set of all representative tropomyosin states without crossbridges, i.e. one state for each of the 3010 classes of states.

# Force calculation and Hill curve fitting procedure

Previous work has repeatedly shown that force-calcium curves do not follow a single Hill curve, but may be reasonably approximated by a biphasic Hill curve with higher cooperativity ($n_H \geq 8$) at lower compared to higher calcium ($n_H \approx 3$–$4$) [52,53].

We have followed this approach throughout our manuscript, and fit $n_2, n_1$ to lower and upper halves of the force-calcium relationship separately for regions below and above $\text{Ca}_{50}$, respectively.

Specifically, we fit the best linear function

$$\log_{10}\left(\frac{F_n}{1-F_n}\right) = n_i\left(\log_{10}[\text{Ca}^{2+}]\right) + b \tag{17}$$

Where the normalized force is:

$$F_n([\text{Ca}^{2+}]) = \frac{F([\text{Ca}^{2+}])}{F(1000\,\mu\text{M})} \tag{18}$$

This fitting is performed in a 'window' of $[\text{Ca}^{2+}]$ within pCa 0.4 from $\text{Ca}_{50}$, to approximate the window shown experimentally [52] as the curve significantly deviates from linear for the extreme lower and upper regions. However, results were obtained for $\text{pCa} = -{}^{10}\log[\text{Ca}^{2+}]$ spaced 0.01 apart between pCa=3 and pCa=12 to ensure Hill fits are possible for a wide range of $K_{\text{DA}}$ and $K_{\text{DI}}$. When reporting a single Hill coefficient we use the mean value of the upper and lower halves, $n_H = (n_1 + n_2)/2$.

For this procedure the force $F$ does not have to be determined, as we know it is proportional to the number of crossbridges bound, which we introduce as $\chi(Ca^{2+})$:

$$F \sim \chi(Ca^{2+}) = \sum_{i=0}^{n}\sum_{j=0}^{m} j \cdot P(N_u(\text{tm}) = i \wedge N_{\text{xb}}(\text{tm}) = j) = \sum_{i=0}^{n}\sum_{j=0}^{m} j \cdot \text{TmXB}_{\text{i,j}} \tag{19}$$

This number can be converted to force by scaling it to a target value $T_{\text{ref}}$ at maximal activation.

$$F = T_{\text{ref}}\frac{\chi([\text{Ca}^{2+}])}{\chi_{\text{max}}} \tag{20}$$

Where $\chi_{\text{max}}$ can be determined by simulating the model at very high $[\text{Ca}^{2+}]$, or well approximated as $\chi_{\text{max}} \approx 0.25 \cdot 69$ based on 69 crossbridges and our parametrization to a duty ratio of approximately 25% at maximal activation. As mentioned in section , we use $T_{\text{ref}} = 120$ kPa as in previous work [35,36].

# Experimental data for rat and human calcium transients

Rat calcium transients were obtained as previously described [62]. Briefly, cardiomyocytes were enzymatically isolated [66], loaded with fluo-4 AM and field stimulated at 6 Hz and 37 °C. $\text{Ca}^{2+}$ transient measurements were recorded by whole-cell photometry. Calcium transients obtained showed significant variability, with a population showing high (greater than 1 µM peak $\text{Ca}^{2+}$, c.f. [67]), and others being more similar to calcium

transients previously measured in mouse (peak $[Ca^{2+}]$approximately 0.5 µM, c.f. [65]). For this study we used a calcium transient representative of the first type to widen the range of model responses tested, as we already include a calcium transient from the mouse.

Human calcium transients were based on data on time to peak, relaxation, size and diastolic levels of the calcium transient from Coppini et al. [68]. Specifically, we used a diastolic calcium $\mathrm{Ca_{dia}} = 0.1399$ µM, a transient magnitude $\mathrm{Ca}_\Delta = 0.3431$ µM, a time to peak of TPT = 48.2 ms, a time to 50% relaxation of $\mathrm{RT}_{50} = 175.9$ ms, and a time to 90% relaxation of $\mathrm{RT}_{90} = 343.1$ ms. The calcium transient was constructed from this experimental data using the formula proposed by Hunter et al. [69].

$$f_{\mathrm{Ca}}(\tau, t) = \mathrm{Ca_{dia}} + \mathrm{Ca}_\Delta \frac{t}{\tau} e^{1-t/\tau} \tag{21}$$

As a single $\tau$ can not match experimental data, we use a piecewise, $C_0$ continuous parametrization defined by:

$$[\mathrm{Ca}^{2+}](t) = \begin{cases} f_{\mathrm{Ca}}(t, \mathrm{TPT}) & t < \mathrm{TPT} \\ f_{\mathrm{Ca}}(t - t_1, \tau_1) & \mathrm{TPT} < t < \mathrm{RT}_{50} \\ f_{\mathrm{Ca}}(t - t_2, \tau_2) & \mathrm{RT}_{50} < t < 2\mathrm{RT}_{90} \\ a - bt & 2\mathrm{RT}_{90} < t < 1000 \end{cases} \tag{22}$$

Where $\tau_1$ is fitted to result in a transient which matches experimental $\mathrm{RT}_{50}$, $\tau_2$ is fitted to result in a transient which matches experimental $\mathrm{RT}_{90} - \mathrm{RT}_{50}$, and $t_1, t_2, a, b$ are chosen to ensure $C_0$ continuity including $[\mathrm{Ca}^{2+}](1000) = [\mathrm{Ca}^{2+}](0)$.
