# Supplementary figures and images for: A Spatially Detailed Model of Isometric Contraction Based on Competitive Binding of Troponin I Explains Cooperative Interactions between Tropomyosin and Crossbridges
Source: PLoS Comput Biol. 2015 Aug 11;11(8):e1004376. doi: 10.1371/journal.pcbi.1004376 (PMC4532474; doi:10.1371/journal.pcbi.1004376)

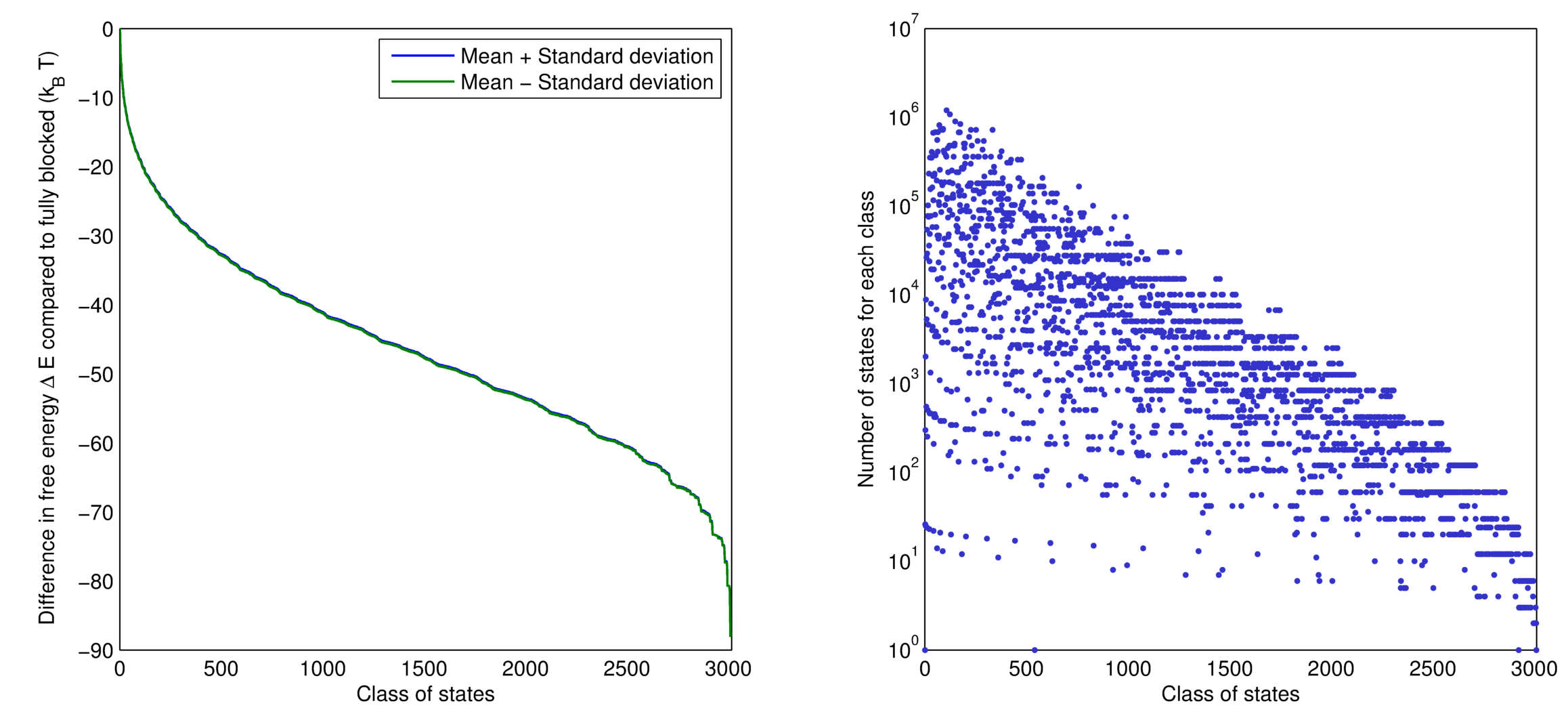

Supplement: S2 Fig — The left plot shows the variation in free energy within each representative class, which is at most 0.11%. The right plot shows the number of states within each class, varying from 1 to 1.2 million. There is a clear tendency for classes with lower energy to have fewer states in each class, as these correspond to longer connected unblocked regions, leading to fewer possible locations within 26 RU. However, due to the symmetries in certain states, classes with a low number of states appear along the entire energy range. Notably, other than the ‘fully blocked’ and ‘fully unblocked’ states, which each have their own class, so do the two additional stats UUUUUUUUBUUUUUUUUBUUUUUUUU, and UUBUUBUUBUUBUUBUUBUUBUUBUU, due to this kind of symmetry. (TIF) [file pcbi.1004376.s003.tif]
